# Drp1-dependent remodeling of mitochondrial morphology triggered by EBV-LMP1 increases cisplatin resistance

**DOI:** 10.1038/s41392-020-0151-9

**Published:** 2020-05-20

**Authors:** Longlong Xie, Feng Shi, Yueshuo Li, We Li, Xinfang Yu, Lin Zhao, Min Zhou, Jianmin Hu, Xiangjian Luo, Min Tang, Jia Fan, Jian Zhou, Qiang Gao, Weizhong Wu, Xin Zhang, Weihua Liao, Ann M. Bode, Ya Cao

**Affiliations:** 1 Key Laboratory of Carcinogenesis and Invasion, Chinese Ministry of Education, Department of Radiology, Xiangya Hospital, Central South University, Changsha, 410078 China; 2 Cancer Research Institute and School of Basic Medical Science, Xiangya School of Medicine, Central South University, Changsha, 410078 China; 3 Key Laboratory of Carcinogenesis, Chinese Ministry of Health, Changsha, 410078 China; 4 Molecular Imaging Research Center of Central South University, Changsha, 410008 Hunan China; 5 Key Laboratory for Carcinogenesis and Cancer Invasion, Chinese Ministry of Education, Zhongshan Hospital, Shanghai Medical School, Fudan University, Shanghai, 200000 China; 6 Department of Otolaryngology Head and Neck Surgery, Xiangya Hospital, Central South University, Changsha, 410078 China; 7 Department of Radiology, Xiangya Hospital, Central South University, Changsha, 410078 China; 8 The Hormel Institute, University of Minnesota, Austin, MN 55912 USA; 9 Research Center for Technologies of Nucleic Acid-Based Diagnostics and Therapeutics Hunan Province, Changsha, 410078 China; 10 National Joint Engineering Research Center for Genetic Diagnostics of Infectious Diseases and Cancer, Changsha, 410078 China

**Keywords:** Head and neck cancer, Drug development, Cancer microenvironment

## Abstract

Latent membrane protein 1 (LMP1) is a major Epstein–Barr virus (EBV)-encoded oncoprotein involved in latency infection that regulates mitochondrial functions to facilitate cell survival. Recently, mitochondrial fission has been demonstrated as a crucial mechanism in oncovirus-mediated carcinogenesis. Mitochondrial dynamin-related protein 1 (Drp1)-mediated mitochondrial fission has an impact on the chemoresistance of cancers. However, the mechanism by which oncogenic stress promotes mitochondrial fission, potentially contributing to tumorigenesis, is not entirely understood. The role of Drp1 in the oncogenesis and prognosis of EBV-LMP1-positive nasopharyngeal carcinoma (NPC) was determined in our study. We show that EBV-LMP1 exhibits a new function in remodeling mitochondrial morphology by activating Drp1. A high level of p-Drp1 (Ser616) or a low level of p-Drp1 (Ser637) correlates with poor overall survival and disease-free survival. Furthermore, the protein level of p-Drp1 (Ser616) is related to the clinical stage (TNM stage) of NPC. Targeting Drp1 impairs mitochondrial function and induces cell death in LMP1-positive NPC cells. In addition, EBV-LMP1 regulates Drp1 through two oncogenic signaling axes, AMPK and cyclin B1/Cdk1, which promote cell survival and cisplatin resistance in NPC. Our findings provide novel insight into the role of EBV-LMP1-driven mitochondrial fission in regulating Drp1 phosphorylation at serine 616 and serine 637. Disruption of Drp1 could be a promising therapeutic strategy for LMP1-positive NPC.

## Introduction

Mitochondria are multifunctional organelles and perform functions, including regulation of cell bioenergetics, calcium homeostasis, reduction-oxidation balance, and programmed cell death. Dysregulation of mitochondria is closely related to tumor initiation and progression.^1^ Mitochondrial dynamics are equilibrated by the opposing processes of fission and fusion in response to the microenvironment. The proteins that participate in fission and fusion regulation are deemed “mitochondria-shaping” proteins, including dynamin-related protein 1 (Drp1), and mitofusins 1 and 2 (Mfn1 and Mfn2, respectively).^2^ Drp1 is required for mitochondrial fission and acts as a protein marker for mitochondrial dynamics. The contribution of active Drp1 is one of the important factors that affect the function of mitochondria.^3^ Strikingly, phosphorylation of Drp1 plays a crucial role in Drp1 activity regulation.^4^ Accumulating evidence indicates that Ser616 phosphorylation promotes the translocation of Drp1 from the cytosol to the mitochondrial outer membrane. However, Ser637 phosphorylation reverses this process.^5^ Drp1 is phosphorylated by specific kinases and signaling pathways during mitochondrial fission, including phosphoglycerate mutase family member 5,^6^ AMPK,^7–9^ MAPK,^10–12^, and cyclin-dependent kinase 1/cyclin B1 (Cdk1/cyclin B1).^13^ AMPK is not only an important detector of energy levels but is also a negative moderator of glycolysis.^14^ In tumors, stress signals (aerobic glycolysis, inflammatory factors, ROS, and hypoxia) can drive mitochondrial fission.^15^ Drp1 participates in all the stages of the cell cycle to produce the energy necessary for basic cellular functions.^16^ Overexpression of cyclin B1 promotes the G2/M phase transition, resulting in uncontrolled cell proliferation and even malignant transformation.^17^

Mitochondria have a unique function in virus-related tumorigenesis. Epstein–Barr virus (EBV) is an important oncovirus whose infection is widespread throughout the world and is related to a variety of cancers, including nasopharyngeal carcinoma (NPC). One of the key oncogenic proteins encoded by EBV is latent membrane protein 1 (LMP1), which is expressed in almost all primary NPC tissues.^18^ LMP1 can simulate CD40 self-oligomerization and dysregulate key cellular processes, causing persistent activation to inhibit host cell death. This oncoprotein plays a significant role in mediating multiple tumorigenic signals that are essential for EBV-mediated transformation.^19^ Previous studies have shown that EBV not only mediates mitochondrial DNA replication^20^, but also modulates DNMT1 mitochondrial localization to inhibit OXPHOS and increase aerobic glycolysis.^21^ Insights from these investigations suggested that EBV was significant in mitochondria-based carcinogenesis. Recently, several researchers have attempted to demonstrate the promoting effects of various oncogenes and viruses on mitochondrial fission in tumors stimulated by kinases.^10,22–26^ However, the mechanism by which oncogenic stress promotes mitochondrial fission, potentially contributing to tumorigenesis, is not entirely understood.

Our study has provided novel insight into a mechanism by which EBV-LMP1 mediates the imbalance of Drp1 phosphorylation at Ser616 and Ser637 through the AMPK and Cdk1 kinase axes. These two phosphorylation sites are closely related to the overall survival and disease-free survival of NPC patients. Importantly, LMP1-mediated mitochondrial fission promotes a metabolic shift to glycolysis. Moreover, preclinical data showed that disruption of oncogenic signaling axes, the AMPK*α* (Thr172)/p-Drp1 (Ser637) or cyclin B1/Cdk1/p-Drp1 (Ser616) pathways by metformin or cucurbitacin E, respectively, significantly increased the sensitivity of NPC cells to cisplatin. These findings provide guidance for the development of therapeutic interventions for EBV-LMP1-positive NPC in the future.

## Results

### The activity of Drp1 is strongly associated with EBV-LMP1 expression in NPC patients

**Fig. 1:**
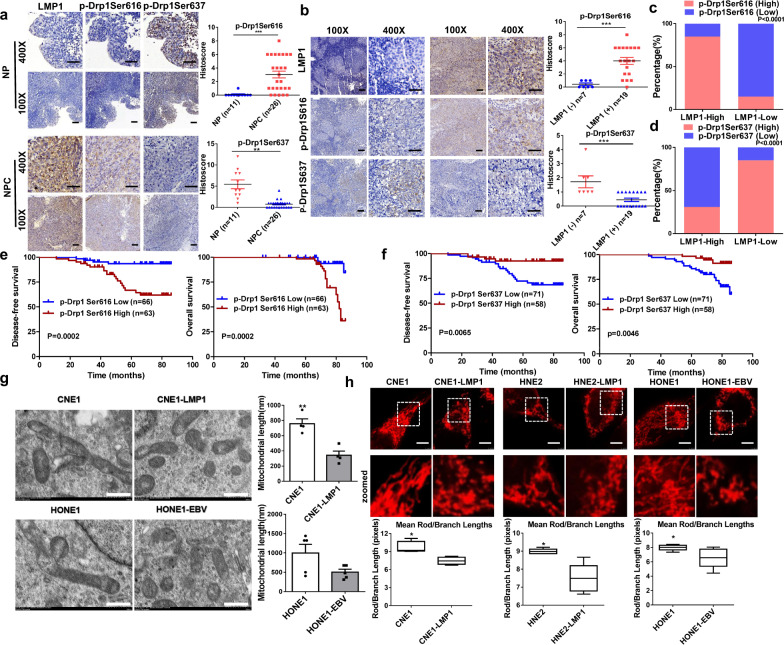
EBV-LMP1 is positively correlated with Drp1 activation in human NPC patient tissues and prominently changes mitochondrial morphology. **a** Representative IHC staining of p-Drp1 Ser616 and p-Drp1 Ser637 levels from tissue slices of 26 nasopharyngeal squamous cell carcinoma patients and 11 nasopharyngitis patients (100×: scale bar, 100 μm; 400×: scale bar, 50 μm; ***P < 0.001 by Student’s *t*-test). **b** Representative IHC photos for the expression of LMP1 and p-Drp1 Ser616 and p-Drp1 Ser637 in NPC tissues (***P < 0.001). **c** The percentage of specimens displaying low or high LMP1 and p-Drp1 Ser616 (***P < 0.001). **d** The percentage of specimens displaying low or high LMP1 and p-Drp1 Ser637 (**P < 0.01). **e** Disease-free survival (left) and overall survival (right) analysis according to p-Drp1 Ser616 expression. The tissue microarray of NPC patients was divided into two groups: good prognosis (low expression of p-Drp1 Ser616) and poor prognosis (high expression of p-Drp1 Ser616). **f** Disease-free survival (left) and overall survival (right) analysis according to p-DRP1 Ser637 expression. The NPC patient tissues in the microarray were divided into two groups: good prognosis (high expression of p-Drp1 Ser637) and poor prognosis (low expression of p-Drp1 Ser637). **g** Transmission electron microscopy photomicrographs of mitochondrial structure in EBV-LMP1-positive and EBV-LMP1-negative cells. Scale bar, 500 nm. Quantification of mitochondrial length is shown in bar graphs. **h** Confocal microscopy analysis of mitochondrial morphology. Red: MitoTracker Red. Images were analyzed using ImageJ software (scale bar, 2.5 μm, *P < 0.05)

The Gene Expression Omnibus (GEO) database was used to examine the mRNA levels of DNM1L (the gene encoding Drp1), Mfn1, and Mfn2 in a cohort of NPC patients (GDS3341). NPC tumor tissues exhibited relatively high *DNM1L* mRNA expression compared to nasopharyngitis tissues (Supplementary Fig. 1a). Moreover, clinical head and neck squamous carcinoma samples from The Cancer Genome Atlas (TCGA) database indicated that patients with low expression of *DNM1L* had better overall survival than patients with high DNM1L expression (Supplementary Fig. 1b). EBV-encoded oncoproteins (such as LMP1) are known to be involved in various mechanisms of NPC tumorigenesis.^18^ These findings encouraged us to explore the roles of the oncoprotein LMP1 in regulating mitochondrial fission in NPC. First, we found that EBV-LMP1 had no significant effect on the expression of the mitochondrial fission protein Drp1 in 26 NPC tissues and 11 nasopharyngitis tissues (Supplementary Fig. 1c). As indicated before, the phosphorylation of Drp1 plays an important role in the regulation of Drp1 activity.^4^ To determine the association between LMP1 and Drp1 activity, we examined the levels of p-Drp1 and LMP1 in these tissues. The clinical characteristics of each patient are listed in Supplementary Table 1. Immunohistochemistry showed that p-Drp1 (Ser616) was highly expressed in NPC tissues, whereas p-Drp1 (Ser637) expression in NPC tissues was decreased compared with that in nasopharyngitis tissues (Fig. 1a), and these effects were associated with LMP1 (Fig. 1b). The protein expression of LMP1 was positively correlated with the level of p-Drp1 (Ser616*; P* = 0.0029; Fig. 1c, Supplementary Table 2) and negatively correlated with the protein level of p-Drp1 (Ser637; *P* = 0.002; Fig. 1d, Supplementary Table 3). These results indicate that the activity of Drp1 appears to be enhanced by the expression of LMP1 in NPC patients. Moreover, the NPC patients with a high level of p-Drp1 (Ser616) exhibited shorter disease-free survival (*n* = 129, median survival time = 53 months vs. 74 months) and overall survival (*n* = 129, median survival time = 70 months vs. 74 months) than the patients with lower levels of p-Drp1 (Ser616; Fig. 1e). In contrast, a high level of p-Drp1 (Ser637) in NPC patients correlated with a longer disease-free survival (*n* = 129, median survival time = 72 months vs. 66 months) and overall survival (*n* = 129, median survival time = 73 months vs. 69 months) than a low level of p-Drp1 (Ser637) according to results from a commercially available tissue microarray (Fig. 1f). Furthermore, we found that age, sex, and cervical lymph node metastasis were independent of Drp1 activity (Supplementary Table 4). Importantly, an advanced clinical stage of the tumor (TNM > 2) was significantly associated with a high level of p-Drp1 (Ser616; *P* = 0.0002, Supplementary Fig. 2a), but clinical stage was not associated with the level of p-Drp1 (Ser637; Supplementary Fig. 2b).

### EBV-LMP1 enhances mitochondrial fission

Drp1 is the crucial executor of mitochondrial fission. Electron microscopic images of NPC cells showed that the number of mitochondria was relatively high in EBV-LMP1-positive cells compared to that in EBV-LMP1-negative cells (Fig. 1g). The results from laser confocal microscopy (Fig. 1h) indicated that the mitochondrial fission ratio of LMP1-positive cells was higher than that of LMP1-negative cells. Quantification of the mean mitochondrial length on the images revealed a significantly shorter mitochondrial length in EBV-LMP1-positive cells than in EBV-LMP1-negative cells (Fig. 1h). Moreover, compared with LMP1-negative NPC cells, cells with enhanced expression of LMP1 had more fragmented mitochondria (Supplementary Fig. 3a), and this morphology was notably changed after transfection with small interfering RNA (siRNA) against LMP1 (Supplementary Fig. 3b). Overall, the levels of phosphorylated protein were consistent with the morphological results and provided strong evidence for the role of LMP1 in the regulation of mitochondrial morphology.

### Imbalance in mitochondrial fission leads to Drp1-mediated NPC cell death

**Fig. 2:**
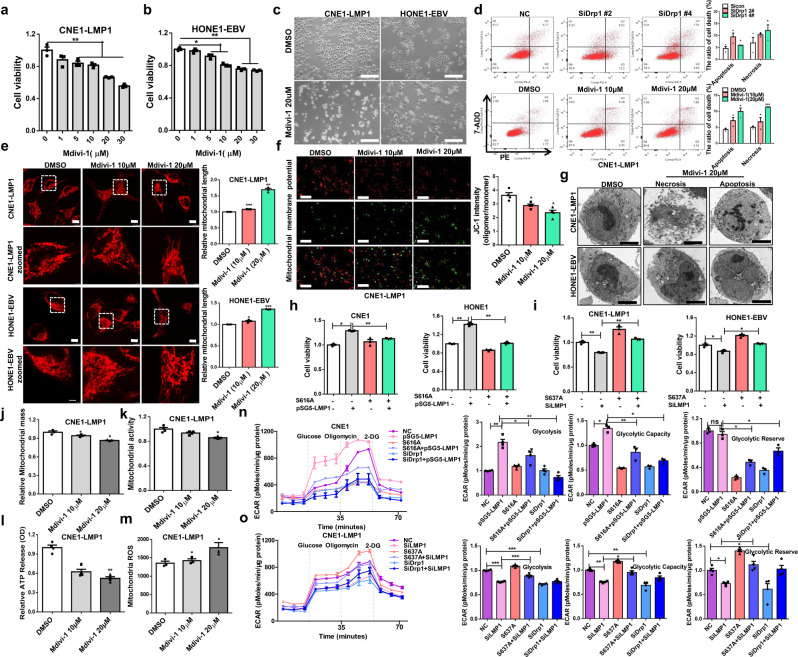
Inhibition of Drp1 activity impairs mitochondrial function and leads to cell death. **a**, **b** The cells were treated with different concentrations of Mdivi-1 or DMSO control for 24 h. Cell viability was analyzed by MTS assay (*P < 0.05, **P < 0.01). **c** Morphology of NPC cells treated with Mdivi-1 or DMSO (scale bar, 100 μm). **d** Flow cytometry analysis of cell death in NPC cells with Drp1 knockdown or Mdivi-1 treatment (*P < 0.05, **P < 0.01). **e** Mitochondrial morphology of CNE1-LMP1 and HONE1-EBV cells treated with or without Mdivi-1 (20 μM) and stained with MitoTracker Red (scale bar, 10 μm). **f** The mitochondrial potential was detected through JC-1 staining. Red fluorescence, which indicated normal mitochondrial potential, was converted into green fluorescence after a reduction in mitochondrial potential. Scale bar, 5 μm. Data are presented as the mean ± S.E.M. (*n* = 3, *P < 0.05). **g** Transmission electron microscopy analysis of photomicrographs of CNE1-LMP1 and HONE1-EBV cells treated with DMSO or Mdivi-1 (scale bar, 5 μm.) **h** MTS assay analysis of the viability of CNE1 and HONE1 cells with CRISPR/Cas9-mediated endogenous modification of Drp1 S616A. **i** MTS assay analysis of the viability of CNE1-LMP1 and HONE1-EBV cells with CRISPR/Cas9-mediated endogenous modification of Drp1 S637A. **j**–**m** CNE1-LMP1 cells were treated with or without Mdivi-1 (10 μM or 20 μM) for 24 h or transfected with siNC or siDNM1L for 48 h. Flow cytometry was performed to determine mitochondrial mass using MitoTracker Green FM (M7514) in NPC cells **j**. The relative fluorescence intensity of cancer cells stained with MitoTracker Red (M7512) was measured to examine the changes in mitochondrial activity **k**. Effects of Drp1 on adenosine triphosphate (ATP) **l**. To assess mitochondrial ROS, cells were stained with MitoSOX, and relative signal intensities were analyzed using flow cytometry **m**. Values represent the mean ± SD of three independent experiments (*P < 0.05, **P < 0.01, ***P < 0.001). **n**, **o** The ECAR was determined using a Seahorse XF96 analyzer to evaluate glycolytic flux. Glycolysis, glycolytic capacity, and glycolytic reserve were determined by the sequential addition of 10 mM glucose, 1 PM oligomycin, and 50 mM 2-D-glucose. Values represent the mean ± SD of four experiments performed five times (*P < 0.05, **P < 0.01, ***P < 0.001). Values were normalized to cell number. S616A-mutated or Drp1‐knockdown CNE1 cells transfected with pSG5-LMP1 **n**. S637A-mutated or Drp1‐knockdown CNE1-LMP1 cells transfected with LMP1 siRNA **o**

Mdivi-1 is considered to be a small-molecule inhibitor of mitochondrial fission that specifically targets Drp1. It inhibits the formation of finger ring-like structures of Drp1 oligomers and suppresses the activity of GTPases.^27^ To investigate whether EBV-LMP1 promotes mitochondrial division and maintains cell survival by increasing Drp1 activity, we measured cell viability by MTS assay after cells were treated with different concentrations of Mdivi-1 for 24 h. The results indicated that Mdivi-1 exhibited a dose-dependent inhibitory effect on NPC cell proliferation (Fig. 2a, b). These results were validated by the EdU incorporation assay (Supplementary Fig. 5a, b). Interestingly, compared to that in the DMSO-treated group, we found that the mean nuclear intensity (CV%) was increased in NPC cells treated with Mdivi-1 (Supplementary Fig. 5c). In addition, the mean nuclear area and roundness of the nuclei decreased significantly (Supplementary Fig. 5d, e). These results suggest that the cells were on the verge of death after mitochondrial fission was inhibited. Additionally, we observed that the appearance of NPC cells became round and transparent with Mdivi-1 treatment (20 μM) compared to that of the untreated control cells (Fig. 2c), suggesting that Mdivi-1 dramatically augments NPC cell membrane permeability. This result matched well with JC-1 staining, which reflects the mitochondrial membrane potential (Fig. 2f). Additionally, Mdivi-1- and SiDrp1-induced cell death was further supported using an apoptosis kit for flow cytometry analysis (Fig. 2d, Supplementary Fig. 4). The fission of mitochondria was reduced with Mdivi-1 treatment (Fig. 2e). Subsequently, transmission electron microscopy was performed to evaluate the ultrastructural features of CNE1-LMP1 and HONE1-EBV cells under Mdivi-1 treatment. However, compared with the DMSO-treated group, these two cell types displayed necrotic (extensive cytoplasmic membrane rupture and leakage of intracellular contents) and apoptotic morphology (nuclear pyknosis with an integrated cytoplasmic membrane) in response to Mdivi-1 treatment (Fig. 2g). These data suggested that mitochondrial fission, which was repressed by Mdivi-1, led to cell death, including both necrosis and apoptosis. Nec-1 and zVAD are specific inhibitors of necroptosis and apoptosis, respectively. To further study the role of Drp1 in cell death, EBV-LMP1-positive NPC cells were treated with Nec-1 and zVAD for 30 min, treated with Mdivi-1 for 24 h, and then subjected to the MTS assay (Supplementary Fig. 6a). The results showed that both Nec-1 and zVAD prevented a substantial amount of cell death following treatment with the indicated concentration of Mdivi-1 (Supplementary Fig. 6b, c). These results indicated that Mdivi-1 might induce both necroptosis and apoptosis in NPC cells by targeting Drp1. Additionally, we found that the cell viability in LMP1-overexpressing CNE1 and HONE1 cells was increased compared with that in control cells, and the effects were suppressed by Drp1 S616A (Fig. 2h). In addition, a significant reduction in cell viability was observed by silencing LMP1 in CNE1-LMP1 and HONE1-EBV cells, but this effect was removed by Drp1 S637A (Fig. 2i). Based on these results, promotion of Drp1 phosphorylation at Ser616, and suppression of Drp1 phosphorylation at Ser637 are required for LMP1-enhanced cell viability and evasion of cell death.

### Drp1 is indispensable for maintaining mitochondrial function

Fragmented mitochondria will contribute to a high mitochondrial mass (Supplementary Fig. 7a) and activity (Supplementary Fig. 7b). We found that mitochondrial mass and activity decreased in EBV-LMP1-positive NPC cells treated with Mdivi-1 or expressing siRNA against Drp1 compared with the respective values in control cells (Fig. 2j, k, Supplementary Fig. 7e, f). Classically, the execution of cell death is thought to involve sharp fluctuations in ROS and ATP levels.^28^ Here, we examined whether ROS and ATP participate in cell death modulated by Drp1 depletion. The results revealed that the concentration of cellular ATP was repressed after treatment with the Drp1 inhibitor Mdivi-1 or expression of siDrp1 in EBV-LMP1-positive NPC cells (Fig. 2l, Supplementary Fig. 7g). Notably, mitochondrial ROS were elevated under the same conditions (Fig. 2m, Supplementary Fig. 7h). These results indicated that targeting Drp1 impaired the mitochondrial functions required for cancer cell survival. Moreover, LMP1-positive cells with high levels of mitochondrial fission had increased glycolytic flux (Supplementary Fig. 7c, d). However, LMP1 failed to rescue the rate of glycolysis, glycolytic capacity, and glycolytic reserve with Drp1 silencing. Similarly, Ser616 mutation (to Ala) weakened the LMP1-Drp1-induced glycolysis flux (Fig. 2n). After mutation of S637 (to Ala) in CNE1-LMP1 cells, the glycolysis flux could not be reduced by LMP1 knockout (Fig. 2o). Taken together, these results show that glycolysis is closely related to mitochondrial fission, which depends on the phosphorylation of Ser616 and the dephosphorylation of Ser637 in EBV-LMP1-positive cells. The enhancement of glycolysis may be a crucial adaptive function of cancer cells under the high level of mitochondrial fission induced by EBV infection stress.

### EBV-LMP1 promotes Drp1 migration from the cytoplasm to mitochondria by activating Drp1

**Fig. 3:**
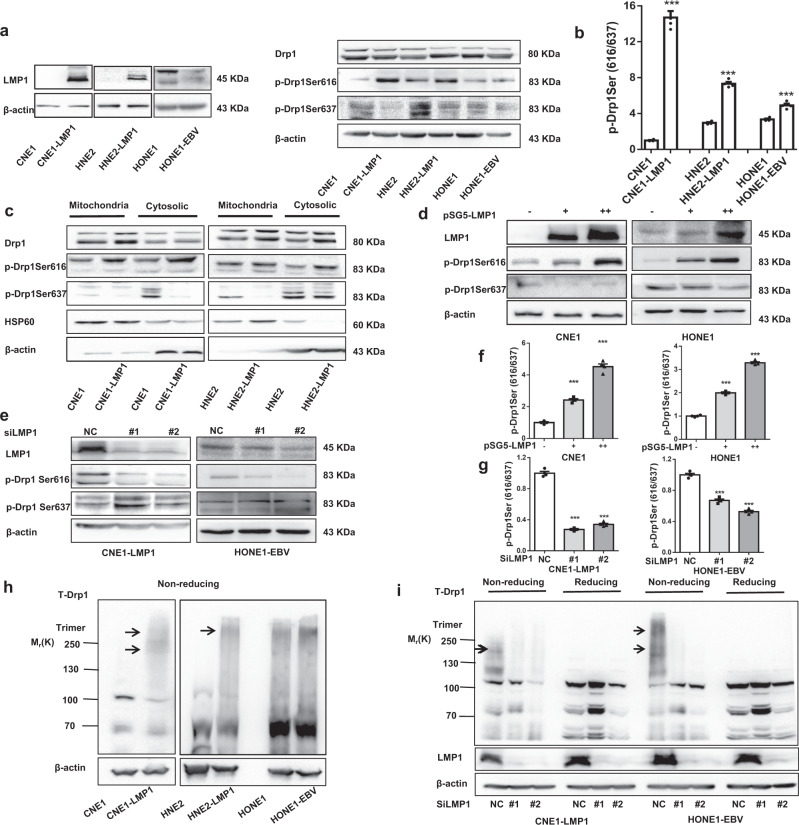
EBV-LMP1 activates the mitochondrial fission protein Drp1. **a**, **b** The effect of LMP1 on Drp1 phosphorylation. NPC cell lysates were subjected to western blot (WB) analysis with the antibodies indicated **a**. The expression ratio of p-Drp1 Ser616 to p-Drp1 Ser637 was calculated via densitometric analysis of each immunoblot using ImageJ software **b**. **c** Subcellular fractions were isolated from NPC cell lines and subjected to WB analysis. **d**, **e** Cell lysates of EBV-LMP1 overexpression or knockdown cell lines were subjected to WB for measurement of the phosphorylation of Drp1 Ser616 and Drp1 Ser637. **f**, **g** The expression ratio of p-Drp1 Ser616 to p-Drp1 Ser637 was calculated via densitometric analysis of each immunoblot using ImageJ software. **h**, **i** Drp1 oligomers were resolved from monomers by nonreducing SDS–PAGE, and specific bands are denoted by arrows. Nonreducing SDS–PAGE detected the effect of EBV-LMP1 on Drp1 self-assembly

Next, we investigated the mechanism by which EBV-LMP1 changes mitochondrial morphology. Subcellular translocation occurs when Drp1 is activated by phosphorylation.^5^ We found that phosphorylation at serines 616 and 637 was markedly changed in EBV-LMP1-positive cells (Fig. 3a). Moreover, the ratio of p-Drp1 (Ser616) to p-Drp1 (Ser637) was significantly enhanced by the regulation of EBV-LMP1 (Fig. 3b). We examined the subcellular localization of Drp1 in EBV-LMP1-positive and EBV-LMP1-negative cells, and observed that EBV-LMP1 increased Drp1 mitochondrial localization (Fig. 3c, Supplementary Fig. 8a). More importantly, EBV-LMP1 increased the level of phosphorylation of Drp1 (Ser616) in mitochondrial fractions and decreased the level of p-Drp1 (Ser637) in cytosolic fractions (Fig. 3c). Similarly, a significant enhancement in the mitochondrial localization of p-Drp1 (Ser616) was observed in LMP1-positive cells compared with that in LMP1-negative cells (Supplementary Fig. 8b). Then, we examined whether the expression level of EBV-LMP1 affects the activity of the mitochondrial fission protein Drp1 by overexpressing and knocking down LMP1. The results indicated (Fig. 3d) that gain of LMP1 in CNE1 and HONE1 cells promoted the phosphorylation of Drp1 at Ser616, and attenuated the phosphorylation of Drp1 at Ser637. LMP1 deficiency in CNE1-LMP1 and HONE1-EBV cells reversed these trends (Fig. 3e). The ratio of p-Drp1 (Ser616) to p-Drp1 (Ser637) was markedly increased with EBV-LMP1 overexpression (Fig. 3f) and dramatically decreased with LMP1 silencing (Fig. 3g), suggesting that LMP1 increases Drp1 activity. Additionally, the immunofluorescence assay results demonstrated that CNE1-LMP1 cells had more mitochondrial localization of p-Drp1 (Ser616) than LMP1-depleted cells (Supplementary Fig. 8c). Overall, these results confirmed that EBV-LMP1 might be involved in the mitochondrial fission by regulating the activation of Drp1.

### EBV-LMP1 benefits Drp1 self-assembly

The polymerization of Drp1 is a necessary and rate-limiting step for mitochondrial fission. In fact, intermolecular interactions among Drp1 monomers, and intramolecular interactions between the N-terminal GTP-binding domain and the C-terminal GED domain are dependent on Drp1 self-assembly and functional modulation.^4^ We determined whether EBV-LMP1 could further regulate the self-assembly of Drp1 after phosphorylation-mediated activation. SDS–PAGE analysis of LMP1-positive cells revealed a prominent species at a molecular mass of 170 kDa or 280 kDa representing a polymer (Fig. 3h). Knockdown of LMP1 by siRNA decreased the formation of Drp1 polymers (Fig. 3i), and overexpression of LMP1 reversed this effect (Supplementary Fig. 9a, b). Overall, these results suggest that EBV-LMP1 stimulated Drp1 polymerization in NPC cells, which drives mitochondrial fission. In addition, we found that the increase in Drp1 polymerization induced by LMP1 could be reduced by an endogenous mutation of Drp1 at S616 (to Ala; Supplementary Fig. 9c). Similarly, in CNE1-LMP1 cells, the formation of Drp1 polymers was attenuated when LMP1 was deleted. However, this reduction was blocked after a mutation of Drp1 at S637 (to Ala; Supplementary Fig. 9d). These data demonstrate that LMP1 benefits Drp1 self-assembly by phosphorylation of Drp1 at Ser616 and dephosphorylation of Drp1 at Ser637.

### EBV-LMP1 inhibits AMPK-regulated phosphorylation of Drp1 (Ser637), promoting mitochondrial fission

**Fig. 4:**
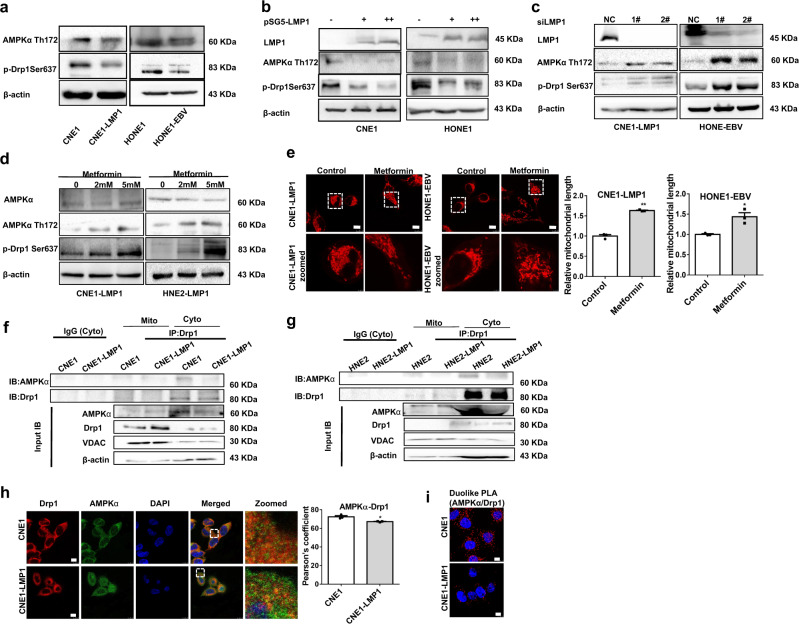
EBV-LMP1 decreases the phosphorylation of Drp1 Ser637 by AMPK. **a** WB analysis of the expression of AMPK*α* Thr172 and p-Drp1 Ser637 in NPC cells. **b**, **c** The protein levels of LMP1, AMPK*α* Thr172, and p-Drp1 Ser637 were detected by WB in LMP1-overexpressing or LMP1-knockdown cells. **d**, **e** LMP1-positive cells were treated with or without metformin (2 mM or 5 mM) for 24 h. WB assays were performed to determine the expression levels of LMP1, AMPK*α* Thr172, and p-Drp1 Ser637 **d**. Confocal microscopy analysis of cell morphology with MitoTracker Red **e**. **f**, **g** Proteins were immunoprecipitated from the mitochondria or cytoplastic lysate and subjected to WB analysis. **h** Confocal microscopy images of CNE1 and CNE1-LMP1 cells immunostained with anti-AMPK*α* (green) and anti-Drp1 (red) antibodies (scale bar, 10 μm). Quantification of the AMPK*α*-Drp1 Pearson’s coefficient is shown in bar graphs (mean ± SD, *P < 0.05 by Student’s *t*-test). **i** The in situ PLA was performed to examine the interaction between AMPK*α* and Drp1 in CNE1, and CNE1-LMP1 cells (scale bar, 10 μm)

Drp1 (Ser637) can be phosphorylated by AMPK.^29^ We previously reported that EBV-LMP1 inhibits AMPK phosphorylation at Thr172.^30^ Consistent with the change in phosphorylated AMPK*α* (Thr172), the phosphorylation of Drp1 (Ser637) was decreased in EBV-LMP1-positive NPC cells (Fig. 4a). Overexpression of LMP1 led to a substantial decline in Drp1 (Ser637) phosphorylation along with decreased AMPK*α* (Thr172) phosphorylation (Fig. 4b). Notably, these results were reversed in the absence of LMP1 (Fig. 4c). Then, we treated cells with metformin, a pharmacological drug that can specifically activate AMPK, at different concentrations (2 or 5 mM). Compared to the untreated control group, the metformin-treated group exhibited high levels of both phosphorylated AMPK*α* (Thr172) and Drp1 (Ser637) in a dose-dependent manner (Fig. 4d). Moreover, we also found that metformin inhibited mitochondrial fission in CNE1-LMP1 and HONE1-EBV cells (Fig. 4e). Taken together, these data suggest that LMP1 activates Drp1 by suppressing the phosphorylation of Drp1 (Ser637), which ultimately promotes mitochondrial fission. Drp1 mainly localizes in the cytoplasm, but when activated, it migrates from the cytoplasm to mitochondria (Supplementary Fig. 8a). Therefore, we assessed the interaction of Drp1 with AMPK at the subcellular level in NPC cells. We extracted mitochondrial and cytoplasmic proteins and observed that the interaction of Drp1 with AMPK was significantly lower in the cytoplasm in LMP1-positive cells than in LMP1-negative cells. However, no direct interaction occurred in the mitochondria (Fig. 4f, g). Additionally, immunofluorescence staining showed that CNE1-LMP1 cells exhibited significant downregulation of the colocalization of Drp1 and AMPK compared to CNE1 cells (Fig. 4h). Additionally, the in situ proximity ligation assay (PLA) showed that EBV-LMP1 significantly decreased interaction between AMPK and Drp1 (Fig. 4i). These results indicate that AMPK interacts with Drp1 in the cytoplasm, phosphorylates Drp1 at Ser637, and inhibits Drp1 migration from the cytoplasm to mitochondria. Overall, we conclude that LMP1 restrains the interaction of Drp1 with AMPK to facilitate mitochondrial fission.

### EBV-LMP1 enhances cyclin B1/Cdk1-mediated mitochondrial fission through Drp1 phosphorylation at Ser616

**Fig. 5:**
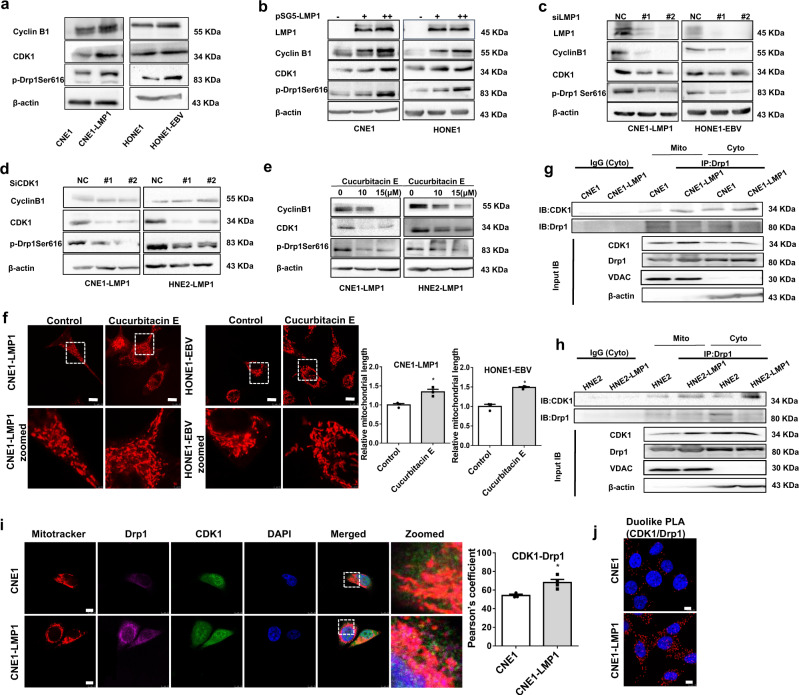
EBV-LMP1 promotes the phosphorylation of Drp1 Ser616 by upregulating cyclin B1/Cdk1 complex activity. **a** WB analysis of the expression of cyclin B1, Cdk1, and p-Drp1 Ser616 in NPC cells. **b**, **c** The protein levels of LMP1, cyclin B1, Cdk1, and p-Drp1 Ser616 were determined by WB analysis in LMP1-overexpressing and LMP1-knockdown cells. **d**, **e** WB was performed to determine the protein levels of cyclin B1, Cdk1, and p-Drp1 Ser616. LMP1-positive cells were treated with or without cucurbitacin E (10 μM) for 24 h **d**. LMP1-positive cells were transfected with siNC or siCDK1 for 48 h **e**. **f** CNE1-LMP1 and HONE1-EBV cells were treated with or without cucurbitacin E (10 μM) for 2 h and stained with MitoTracker Red, which was detected by confocal microscopy (scale bars, 10 μm). **g**, **h** Proteins were immunoprecipitated from the mitochondrial or cytoplasmic lysates to test the interaction of Drp1 and Cdk1. **i** Confocal microscopy images of CNE1 and CNE1-LMP1 cells immunostained with anti-Cdk1 (green) and anti-Drp1 (purple) antibodies, and mitochondria markers (red). The scale bars represent 10 μm. Quantification of the Cdk1-Drp1 Pearson’s coefficient is shown in bar graphs (mean ± SD, *P < 0.05 by Student’s *t*-test). **j** The interaction between Cdk1 and Drp1 in CNE1, and CNE1-LMP1 cells was analyzed by an in situ PLA assay (scale bar, 10 μm)

Cdk1 is the upstream kinase of Drp1 during mitochondrial fission.^13^ We observed that EBV-LMP1 promoted the expression of cyclin B1 and Cdk1, suggesting that EBV-LMP1 activates this complex. We found that compared with those in LMP1-negative cells, the levels of Cdk1 and phosphorylation of Drp1 (Ser616) in EBV-LMP1-positive cells were upregulated (Fig. 5a). Overexpression of LMP1 led to a significant increase in Drp1 (Ser616) phosphorylation with enhanced expression of cyclin B1/Cdk1 (Fig. 5b), and this effect was eliminated after the deletion of LMP1 (Fig. 5c). Knockdown of Cdk1 by siRNA reduced the protein level of phosphorylated Drp1 (Ser616), which was consistent with the changes in the level of cyclin B1/Cdk1 (Fig. 5d). Cucurbitacin E is a specific inhibitor of the cyclin B1/Cdk1 complex and is commonly found in the Cucurbitaceae family, and it has been effective as a therapeutic agent.^31^ Pharmacological blockade of Cdk1 with the specific inhibitor cucurbitacin E reduced the phosphorylation of Drp1 (Ser616; Fig. 5e) and mitochondrial fission (Fig. 5f). Importantly, we found that LMP1 notably enhanced the interaction of Drp1 with Cdk1 in mitochondria and cytoplasm (Fig. 5g, h). These results indicated that Cdk1 not only activates Drp1 in the cytoplasm, phosphorylates Drp1 (Ser616), and promotes the migration of Drp1 from the cytoplasm to mitochondria, but also binds to Drp1 in the mitochondria to form a complex that further increases mitochondrial division. Subsequently, we also observed that dual fluorescent staining of Drp1 and Cdk1 in CNE1-LMP1 cells was significantly higher than that in CNE1 cells (Fig. 5i). Moreover, we used an in situ PLA to further verify that EBV-LMP1 promotes the interaction of these two proteins (Fig. 5j). Taken together, these results suggest that LMP1 enhances Drp1 phosphorylation at Ser616 and promotes mitochondrial fission by activating cyclin B1/Cdk1.

### Metformin and cucurbitacin E enhance the sensitivity of NPC to cisplatin by inhibiting the activity of Drp1 in vitro and in vivo

**Fig. 6:**
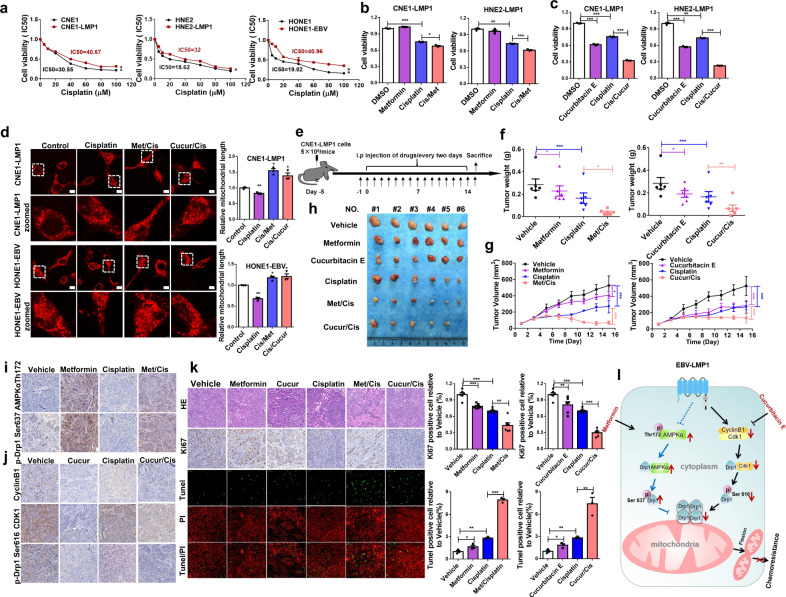
Metformin and cucurbitacin E enhance the sensitivity of NPC to cisplatin by inhibiting the activity of Drp1 in vitro and in vivo. **a** EBV-LMP1-positive and EBV-LMP1-negative cells were treated with increasing concentrations of cisplatin for 24 h. Cell viability was determined by MTS assay. Values are presented as the mean ± S.E.M. from three independent experiments (*P < 0.05; **P < 0.01). **b** CNE1-LMP1 and HNE2-LMP1 cells were treated with cisplatin (20 μM) alone or cisplatin (20 μM) in combination with metformin (5 mM) for 24 h. Cell viability was measured by MTS assay (*P < 0.05; ***P < 0.001). **c** CNE1-LMP1 and HNE2-LMP1 cells were treated with cisplatin (20 μM) alone or cisplatin (20 μM) in combination with cucurbitacin E (10 μM) for 24 h. Cell viability was measured with an MTS assay (***P < 0.001). **d** CNE1-LMP1 and HONE1-EBV cells were treated with control, cisplatin, the combination of cisplatin and metformin, or cucurbitacin E for 5 h. The morphology of mitochondria was analyzed by confocal microscopy (scale bars, 10 μm). **e** The overall diagram of the study design. The nasopharyngeal carcinoma xenograft model was established using CNE1-LMP1 cells. **f**, **g** The tumor weight **f** and tumor volume **g** of CNE1-LMP1-derived xenograft tumors with various treatments. The asterisks indicate a significant difference (*P < 0.05, **P < 0.01, ***P < 0.001). **h** Representative images of xenografts from different treatment groups. **i** Immunohistochemistry staining to determine the protein levels of AMPK*α* Thr172 and p-Drp1 Ser637 in xenograft tumor tissues. **j** Immunohistochemistry to determine the protein levels of cyclin B1, Cdk1, and p-Drp1 Ser616 in representative tumor tissues. **k** Immunohistochemistry to determine the protein levels of Ki67. Apoptosis was analyzed by TUNEL assay. The asterisks indicate a significant difference (*P < 0.05, **P < 0.01, ***P < 0.001). **l** Schematic illustrating that EBV-LMP1 confers chemotherapy resistance by differentially regulating the Drp1 signaling axis. EBV-LMP1 mediates the imbalance of Drp1 phosphorylation at Ser616 and Ser637 via the downstream cyclin B1/Cdk1 and AMPK signaling axes

In previous studies, we demonstrated that EBV-LMP1 confers necroptosis resistance by regulating RIPK1/3 polyubiquitination^32^ or hypermethylation of the RIP3 promoter.^33^ Here, we found that the IC50 of cisplatin in EBV-LMP1-positive cells was significantly higher than that in EBV-LMP1-negative cells (Fig. 6a). Therefore, we determined whether this chemoresistance is associated with the function of EBV-LMP1-mediated mitochondrial fission-related signaling pathways. Our results revealed that metformin or cucurbitacin E combined with cisplatin significantly reduced the cell viability compared to cisplatin alone in LMP1-positive cells (Fig. 6b, c). Furthermore, we observed that mitochondrial fission was increased with cisplatin treatment, and the effect was reversed by metformin or cucurbitacin E treatment (Fig. 6d). To further validate whether metformin and cucurbitacin E sensitize NPCs to cisplatin by regulating Drp1 phosphorylation, we examined the effect of the combination of cisplatin with metformin or cucurbitacin E in an in vivo xenograft mouse model (Fig. 6e). Our results showed that a combination of metformin or cucurbitacin E with cisplatin led to a significant reduction in tumor weight (Fig. 6f) and tumor volume (Fig. 6g, h) compared to vehicle, metformin, cucurbitacin E, or cisplatin treatment alone. The results of western blotting and immunohistochemistry showed an obvious enhancement of AMPK*α* (Thr172) and p-Drp1 (Ser637) levels in the metformin and cisplatin combination group compared to either the vehicle or cisplatin group (Supplementary Fig. 10a, Fig. 6i). We also found a robust reduction in cyclin B1, Cdk1, and p-Drp1 (Ser616) in the cucurbitacin E, and cisplatin combination group (Supplementary Fig. 10b, Fig. 6j). Additionally, the results revealed that the expression of Ki67 was significantly reduced in the metformin or cucurbitacin E and cisplatin combination treatment groups. The TUNEL assay showed that the population of apoptotic cells was enhanced in the combination treatment group (Fig. 6k). A significant loss in mouse body weight (B.W.) was observed in the cisplatin-treated group, but not in the metformin- or cucurbitacin E-treated group (Supplementary Fig. 10c). H&E histological analysis of various organs showed no significant abnormal changes in all groups (Supplementary Fig. 10d). The in vivo study results are in agreement with the in vitro study findings, and indicate that Drp1 is a potential target for decreasing the proliferation and survival of NPC cells. Interfering with the Drp1 upstream kinase AMPK or cyclin B1/Cdk1 via metformin or cucurbitacin E increased the chemosensitivity of NPC tumors to cisplatin.

## Discussion

Cancer cells exhibit high fission mitochondrial morphology and function, which affects tumorigenesis and provides an adaptive environment for their survival.^34^ At present, there are some innovative reports on the relationship between changes in mitochondrial morphology and function in different types of tumors, including glioblastoma,^7^ melanoma,^10^ pancreatic cancer,^22^ lung cancer, breast cancer, NPC, and thyroid tumors.^35–38^ Here, we found that the activity of Drp1 was significantly stronger in EBV-LMP1-positive NPC patients and cells and, importantly, that p-Drp1 (Ser616) was related to the clinical stage of NPC. Moreover, a high level of p-Drp1 (Ser616) or a low level of p-Drp1 (Ser637) correlated with poor overall survival and disease-free survival, indicating that Drp1 is a valuable mitochondrial biomarker in viral infection-related tumorigenesis and determining prognosis. Recent studies have revealed intriguing aspects of how oncoviruses exploit changes in mitochondrial morphology to maintain infection. Hepatitis B virus and hepatitis C virus (HBV and HCV, respectively) enhance the phosphorylation of Drp1 (Ser616), and promote its translocation to mitochondria.^25,26^ Mitochondrial fragmentation is closely related to the regulation of Drp1 by EBV-LMP2A, guiding cell migration and epithelial–mesenchymal transition in gastric cancer and breast cancer cells.^23^ Our data showed that EBV-LMP1 differentially regulates Drp1 phosphorylation at Ser616 and Ser637, significantly increasing mitochondrial fission. This high fission state of mitochondria participates in cytoprotective effects as an adaptive mechanism under a microenvironment of mitochondrial stress induced by EBV infection.

Oncogene-regulated metabolic reprogramming will guide alterations in mitochondrial morphology to sustain metabolic changes. Accumulating evidence shows that Drp1-induced mitochondrial fission is associated with a shift in pro-glycolytic activity.^39^ Moreover, BRAF-V600E-driven melanoma cells have high levels of mitochondrial fission, which enhances glycolytic metabolism.^10^ Consistent with these findings, our results showed that the phosphorylation of Drp1 (Ser616 or Ser637) was indispensable for the enhanced glycolytic metabolism regulated by LMP1. Blocking these high rates of mitochondrial fission in tumor cells will lead to the cell death. Recently, studies revealed that suppression of the activity of Drp1 results in the release of cytochrome c, which leads to apoptosis.^40^ Other studies indicated that inactivation of Drp1 led to programmed necrotic cell death.^41^ TNF-*α* and Mdivi-1 reduce mitochondrial fission and increase necroptosis.^42^ Mdivi-1 induced cell death in vitro, suggesting that Mdivi-1 inhibited mitochondrial fission and suppressed cancer cell survival.^7,37^ Herein, we noted that EBV-LMP1 exerted a strong influence on mitochondrial function by promoting mitochondrial division. Moreover, Mdivi-1 decreased the cell viability of NPC cells, but this reduction was restored by Nec-1 or zVAD treatment. These results suggest that programmed necrosis and apoptosis coexist after Mdivi-1 treatment. However, the detailed mechanism needs to be further studied.

As a crucial molecule in mitochondrial fission, Drp1 is regulated by specific kinases and signaling pathways.^2^ A preliminary study showed that metformin phosphorylated Drp1 (Ser637) by activating AMPK, which hindered the recruitment of Drp1 to the mitochondrial outer membrane and inhibited mitochondrial fission.^29^ Previously, our study demonstrated that EBV-LMP1 significantly inhibited p53-mediated cell cycle arrest, and apoptosis by regulating key proteins and kinases (cyclin D1/Cdk4, cyclin A/Cdk2, and cyclin B1/Cdk1) in NPC cells.^43^ Indeed, the cyclin B1/Cdk1 complex upregulates the phosphorylation of Drp1 (Ser616) and promotes mitochondrial fission, accelerating the progression of cancer.^13^ During mitosis, cyclin B1/Cdk1 phosphorylates Drp1 at Ser616, leading to the mitochondrial translocation of Drp1.^44^ Here, we showed that EBV-LMP1 could regulate Drp1 via two signaling axes: AMPK*α* (Thr172)/p-Drp1 (Ser637) and cyclin B1/Cdk1/p-Drp1 (Ser616). These findings enrich the understanding of the EBV-LMP1 oncogenic signaling network responsible for the regulation of mitochondria.

Mitochondrial fission regulates chemoresistance in human cancers. Drp1-mediated mitochondrial fission is found in metastatic cancer cells and tumor-initiating cells, contributing to the increases in migration and chemoresistance, respectively, in these cells.^7,36,45^ Resveratrol sensitizes NPC cells to 5-FU through the downregulation of the Cox-2/Drp1 signaling axis.^37^ In addition, bone marrow-derived mesenchymal stem cells promote chemoresistance via ERK/Drp1.^39^ Additionally, miR-488 inhibited mitochondrial fission by regulating Six1/Drp1 signaling and reduced chemoresistance to cisplatin.^46^ ROS-regulated mitochondrial fission has been shown to be resistant to cisplatin under hypoxic conditions.^45^ Cisplatin has been the first-line treatment for locally advanced NPC patients during induction chemotherapy and concurrent radiochemotherapy.^47^ The AMPK agonist metformin and the Cdk1-specific inhibitor cucurbitacin E have antitumor effects in multiple cancer cell lines.^30,31^ Several preclinical experiments have shown that there are still many uncertainties regarding the mechanism by which the Drp1-specific inhibitor Mdivi-1 affects cell survival.^48^ In our study, differential disruption of two signaling axes, AMPK*α* (Thr172)/p-Drp1 (Ser637) and cyclin B1/Cdk1/p-Drp1 (Ser616), with metformin and cucurbitacin E enhanced the chemosensitivity of NPC to cisplatin in vivo and in vitro. These data indicate that Drp1 is a novel therapeutic target for overcoming cisplatin resistance and improving the prognosis of NPC patients.

In conclusion, we report that phosphorylation of Drp1 at either serine 616 or serine 637 is a prognostic biomarker in NPC patients. The EBV-encoded latent protein LMP1 differentially regulated two oncogenic signaling axes to elevate mitochondrial fission, which benefited NPC cell survival and chemoresistance (Fig. 6l). This discovery supports the Drp1 phosphorylation sites Ser616 and Ser637, as crucial targets for enhancing chemotherapy sensitivity to cisplatin in NPC in the future.

## Materials and methods

### Cell lines and culture

NPC cell lines (CNE1 and HNE2 cells) were purchased from the Cell Resource Center of Central South University. HONE1/HONE1-EBV cells were generously provided by Professor Sai Wah Tsao from the University of Hong Kong. CNE1-LMP1 and HNE2-LMP1 cells were established by our laboratory and maintained in RPMI-1640 medium (Gibco, USA) supplemented with 10% fetal bovine serum (BI, Israel) and penicillin/streptomycin/gentamicin antibiotics in a humidified incubator with 5% CO_2_ at 37 °C.

### Reagents and antibodies

Metformin, cucurbitacin E, and cisplatin were purchased from MedChemExpress (Cat: HY-17471A, HY-N0417, and HY-17394, respectively, New Jersey, USA). Necrostatin-1 and zVAD-fmk were obtained from Sigma-Aldrich (Cat: N9037 and C2105, respectively, Saint Louis, USA). Antibodies, including anti-p-Drp1 (Ser616), anti-p-Drp1 (Ser637), anti-cyclin B1, anti-AMPK, and anti-AMPK*α* (Thr172) antibodies (Cat: 3455 T, 4867 T, 9870 T, 2532 S, and 2531 S, respectively), were purchased from Cell Signaling Technology (Boston, USA); anti-Drp1, anti-Cdk1, and anti-VDAC1 antibodies (Cat: ab184247, Ab18, and Ab34726, respectively, Abcam, Cambridge, UK); anti-HSP60 antibodies (Cat: Sc-1052, Santa Cruz Biotechnology, Dallas, USA); mouse anti-β-actin antibodies (Cat: A5441, Sigma, Darmstadt, Germany); and Alexa Fluor 488-conjugated goat anti-mouse IgG, Alexa Fluor 647-conjugated goat anti-mouse IgG, and Alexa Fluor 594-conjugated goat anti-rabbit IgG antibodies (Cat: A11001, A21236, and A21207, respectively, Carlsbad, USA) were obtained from Invitrogen.

### Western blot analysis

Cells were harvested and washed twice with phosphate-buffered saline (PBS), lysed with immunoprecipitation (IP) lysis buffer (Thermo Scientific, MA, USA) for 30 min on ice, and centrifuged for 20 min at 12,000 × *g* at 4 °C. Whole-cell lysates were subjected to SDS–PAGE and transferred onto nylon membranes. Membranes were blocked with 5% nonfat milk (or 5% BSA) for 1 h at room temperature, and then incubated with specific primary antibodies at 4 °C overnight. They were then washed with PBS-T three times and subsequently hybridized with peroxidase-conjugated secondary antibodies for 1 h at room temperature, followed by washing with PBS-T. Finally, the ChemiDoc XRS system (Bio-Rad, USA) and Image Lab Software were used for visualization of blots.

### Plasmids and RNA interference

The siRNAs against LMP1, Drp1, and Cdk1 were purchased from Genechem (Shanghai, China), and verified by DNA sequencing. The full-length LMP1 cDNAs were cloned into the GV141 vector (CMV-MCS-3FLAGSV40- Neomycin). The siRNAs and LMP1-overexpressing plasmids were transfected into CNE1-LMP1, HNE2-LMP1, and HONE1-EBV cell lines using Lipofectamine 2000 (Invitrogen, New York, USA) following the manufacturer’s protocol.

### Mitochondrial isolation

The mitochondrial extract was prepared in MSHE + BSA buffer (210 mM mannitol, 70 mM sucrose, 5.0 mM HEPES, 1.0 mM EGTA, 0.5% (w/v) fatty acid-free bovine serum albumin (BSA), and complete protease inhibitor cocktail, pH 7.2). Each sample collected required ~2–4 × 10^7^ cells, which were suspended in 1 ml MSHE + BSA buffer, gently mixed and placed on ice for 5 min. Subsequently, the supernatant fraction was collected by centrifugation following the homogenization of mitochondria (60 times of full grinding at ~2000 rpm for 10 min at 4 °C). The supernatant fraction was obtained by high-speed centrifugation (1200 rpm for 30 min at 4 °C) and contained cytoplasmic proteins. Then, 200 μl of MSHE + BSA buffer was added to the cell pellet, and the mixture was mixed and centrifuged at 1300 rpm for 10 min at 4 °C. The pellet was resuspended in 80 μl of IP lysis buffer and left on ice for 30 min to obtain the mitochondrial proteins.

### Mitochondrial mass and activity

Cells were incubated with MitoTracker (Green or Red) FM probe at a final concentration of 25 nM (M7514 or M7512, Life Technologies) at 37 °C (5% CO_2_) for 15 min. Next, cells were rinsed with PBS three times and digested with 0.25% trypsin solution without EDTA. Finally, cells were collected and suspended in 200 μl of PBS, and the average intensity of fluorescence was analyzed by a Cell Insight™ CX5 imaging system (Operetta, PerkinElmer, USA).

### Immunohistochemical analysis

The study was approved by the Medical Ethics Committee of Xiangya Hospital, Central South University (no. 201803134; Changsha, China). All patients signed informed consent forms for sample collection. A total of 37 paraffin sections were prepared, including 26 samples of NPC tissues and 11 samples of nasopharyngitis tissues obtained from Xiangya Hospital. The NPC tissue array was purchased from Shang Hai (HNasN129Su01, Outdo Biotech Co.) Immunohistochemistry was performed as described previously.^30^ The staining score of each protein was analyzed by two pathologists independently. The immunohistochemistry score depended on the degree of staining and the rate of positive cells. The patients were divided into low and high expression groups according to the median scores of p-Drp1 (Ser616) and p-Drp1 (Ser637).

### Immunofluorescence analysis

Cells were seeded into a chamber slide at a confluence of 30–50% and then incubated with the MitoTracker Red probe (25 nM final concentration) in prewarmed RPMI-1640 medium without FBS and antibiotics. Cells were washed with PBS three times and fixed with 3.7% paraformaldehyde at 37 °C for 20 min. After another PBS wash, cells were blocked with 5% donkey serum for 1 h at room temperature and incubated with primary antibodies at 4 °C overnight. Cells were then hybridized with FITC-conjugated secondary antibodies for 1 h. DAPI was used for counterstaining. Mitochondrial morphology and colocalization coefficients were examined by confocal microscopy (LSM 510 META, Germany) and quantitatively evaluated with ImageJ software.^49^

### Mitochondrial membrane potential and permeability

Cells were seeded in 96-well plates and incubated with the JC-1 probe (5 μM final concentration). The probe was prepared with PBS, and after it was added to cells, they were incubated in the dark at 37 °C for 30 min and then washed three times with PBS. The fluorescence was examined by microscopy (LSM 510 META, Germany) and analyzed using ImageJ software.^50^

### Tumor xenograft studies

The in vivo study was approved by the Medical Ethics Committee (for experimental animals) of Xiangya Hospital, Central South University (no. 201803135). Female BALB/c-nude mice (5–6-week old) were purchased from SLAC Laboratory Animal Co. Ltd. (Changsha, China). All mice were subcutaneously inoculated with CNE1-LMP1 cells (5 × 10^6^ cells/mouse) in the right armpit. When the tumor volume reached 100 mm^3^ (day 6 after injection), the mice were divided randomly into six groups (*n* = 6 each; saline-treated (control), cisplatin-treated, metformin-treated, cucurbitacin E-treated, cisplatin and metformin-treated, and cisplatin and cucurbitacin-treated). Single-drug treatment with metformin (250 mg/kg), cucurbitacin E (0.5 mg/kg), or cisplatin (4 mg/kg) was initiated by i.p. injection. The vehicle control was administered in 0.9% saline. Tumor volume and B.W. were recorded every 2 days. The tumor size was calculated as follows: tumor size = ab^2^/2, where a and b are the larger and smaller diameters, respectively. After 15 days of treatment, the mice were euthanized, and tumors were removed and weighed.

### Database analysis

The GEO database can be accessed online (https://www.ncbi.nlm.nih.gov/geo/). TCGA database (*N* = 502) was used to examine the overall survival of patients according to *DNM1L* levels in head and neck squamous cell carcinoma.

### Statistical analysis

All statistical calculations were performed with the GraphPad Prism 5 software program (GraphPad Software). The experimental data are presented as the mean value ± S.E.M. The statistical significance of the data was analyzed using ANOVA or a standard Student’s *t*-test. Overall and disease-free survival were determined by the Kaplan–Meier method and compared using a log-rank test. A *P*-value of <0.05 was deemed statistically significant.

The materials and methods for coimmunoprecipitation, transmission electron microscopy, MTS, EdU incorporation, flow cytometry, ATP and ROS experiments, determination of the extracellular acidification rate (ECAR), TUNEL assays, PLAs, and CRISPR-Cas9 plasmid transfection (mutant Drp1S616A and S637A) are provided in the Supplementary Materials.

## Supplementary information

Supplementary Materials

## Data Availability

The datasets supporting the results of this article are included within the article and its additional files.
