# Correction: Drp1-dependent remodeling of mitochondrial morphology triggered by EBV-LMP1 increases cisplatin resistance

**DOI:** 10.1038/s41392-022-01261-y

**Published:** 2022-12-20

**Authors:** Longlong Xie, Feng Shi, Yueshuo Li, We Li, Xinfang Yu, Lin Zhao, Min Zhou, Jianmin Hu, Xiangjian Luo, Min Tang, Jia Fan, Jian Zhou, Qiang Gao, Weizhong Wu, Xin Zhang, Weihua Liao, Ann M. Bode, Ya Cao

**Affiliations:** 1 Key Laboratory of Carcinogenesis and Invasion, Chinese Ministry of Education, Department of Radiology, Xiangya Hospital, Central South University, Changsha, China; 2 Cancer Research Institute and School of Basic Medical Science, Xiangya School of Medicine, Central South University, Changsha, China; 3 Key Laboratory of Carcinogenesis, Chinese Ministry of Health, Changsha, China; 4 Molecular Imaging Research Center of Central South University, Changsha, Hunan China; 5 Key Laboratory for Carcinogenesis and Cancer Invasion, Chinese Ministry of Education, Zhongshan Hospital, Shanghai Medical School, Fudan University,, Shanghai, China; 6 Department of Otolaryngology Head and Neck Surgery, Xiangya Hospital, Central South University, Changsha, China; 7 Department of Radiology, Xiangya Hospital, Central South University, Changsha, China; 8 The Hormel Institute, University of Minnesota, Austin, MN USA; 9 Research Center for Technologies of Nucleic Acid-Based Diagnostics and Therapeutics Hunan Province, Changsha, China; 10 National Joint Engineering Research Center for Genetic Diagnostics of Infectious Diseases and Cancer, Changsha, China

Correction to: *Signal Transduction and Targeted Therapy* (2020) **5**:56, 10.1038/s41392-020-0151-9, published online 20 May 2020

In this article^1^ an error was noticed in Fig. 3d left (p-Drp1 Ser637). The images were misassigned. And one writing error was found in Fig. S1c. The correct figures are given. The authors confirm that these corrections do not change the result interpretation or conclusions of the article.

**Fig. 3:**
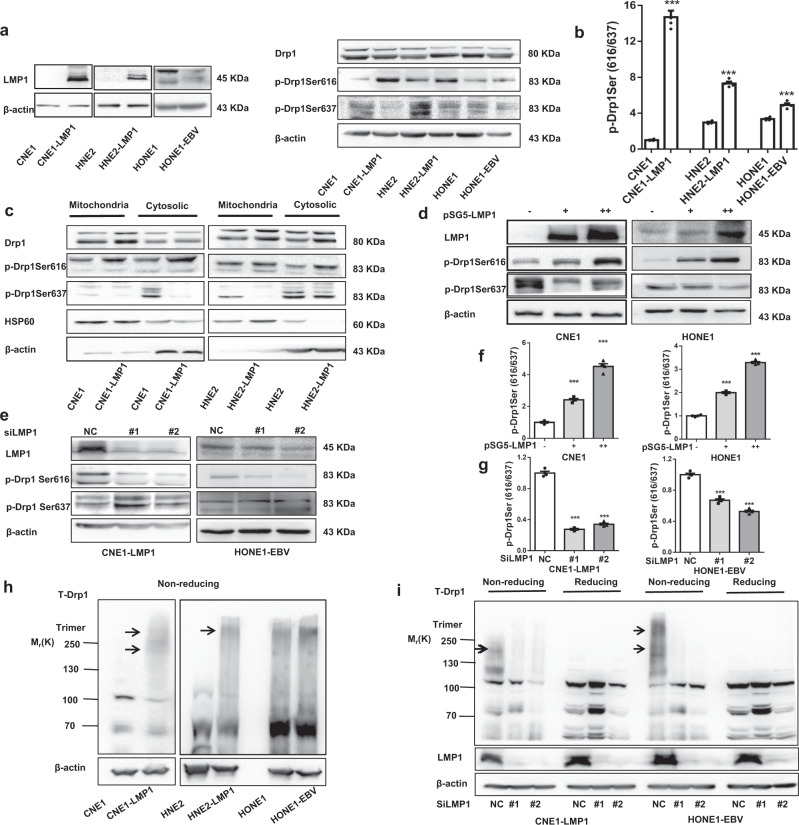
EBV-LMP1 activates the mitochondrial fission protein Drp1. **a**, **b** The effect of LMP1 on Drp1 phosphorylation. NPC cell lysates were subjected to western blot (WB) analysis with the antibodies indicated **a**. The expression ratio of p-Drp1 Ser616 to p-Drp1 Ser637 was calculated via densitometric analysis of each immunoblot using ImageJ software **b**. **c** Subcellular fractions were isolated from NPC cell lines and subjected to WB analysis. **d**, **e** Cell lysates of EBV-LMP1 overexpression or knockdown cell lines were subjected to WB for measurement of the phosphorylation of Drp1 Ser616 and Drp1 Ser637. **f**, **g** The expression ratio of p-Drp1 Ser616 to p-Drp1 Ser637 was calculated via densitometric analysis of each immunoblot using ImageJ software. **h**, **i** Drp1 oligomers were resolved from monomers by nonreducing SDS–PAGE, and specific bands are denoted by arrows. Nonreducing SDS–PAGE detected the effect of EBV-LMP1 on Drp1 self-assembly

**Fig. S1:**
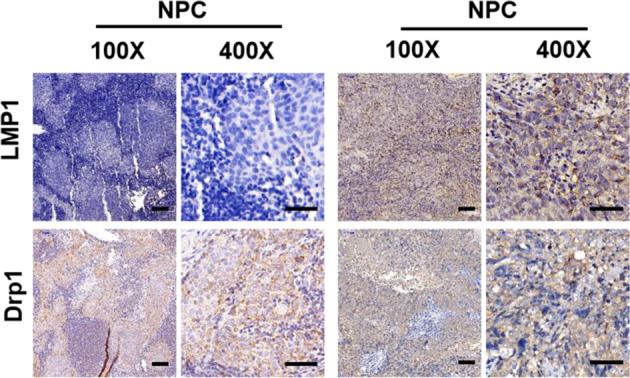
(c) Representative IHC staining of LMP1 and *Drp1* expression from tissues slices of 26 NPC patients. 100x: Scale bar, 100 μm; 400x: Scale bar, 50 μm
