# Supplementary material for: Drp1-dependent remodeling of mitochondrial morphology triggered by EBV-LMP1 increases cisplatin resistance
Source: Signal Transduct Target Ther. 2020 May 20;5:56. doi: 10.1038/s41392-020-0151-9 (PMC7237430; doi:10.1038/s41392-020-0151-9)
Supplement: Supplementary file 1 — Supplementary Materials [file 41392_2020_151_MOESM1_ESM.docx]

Supplementary Materials for

Drp1-dependent remodeling of mitochondrial morphology triggered by EBV-LMP1 increases cisplatin resistance

Longlong Xie^1,2,3^, Feng Shi^1,2,3^, Yueshuo Li^1,2,3^, We Li^1,2,3^, Xinfang Yu^1,2,3^, Lin Zhao ^1,2,3^, Min Zhou^1,2,3^, Jianmin Hu^1,2,3^, Xiangjian Luo^1, 2, 3, 4^, Min Tang^1,2,3, 4^, Jia Fan^5^, Jian Zhou^5^, Qiang Gao^5^, Weizhong Wu^5^, Xin Zhang^6^, Weihua Liao^7^, Ann M. Bode^8^, Ya Cao^1,2, 3,4,9,10^*

Correspondence to: ycao98@vip.sina.com.

**This PDF file includes:**

Materials and Methods

Figures. S1 to S10

Tables S1 to S4

**Materials and Methods**

Co-immunoprecipitation

The magnetic beads (20 μL; Invitrogen, MA, USA) were pre-cleared and mixed with 200 μg pre-cleared mitochondrial protein and 500 μg cytoplasmic protein for 1 h at 4°C. Subsequently, all the pre-cleared samples were incubated with anti-Drp1 or anti-IgG (2 μg/sample) overnight at 4°C. Then 20 μL magnetic beads were added to samples and incubated for 2 h at 4°C. The samples were washed 3 times with cold PBS and boiled with loading buffer for 5 min. Finally, the interaction between antibodies was analyzed by Western blotting.

Transmission electron microscopy

Cells were removed from the culture medium and quickly added to the electron microscopic stationary liquid without any rinsing or digestion. Gently scrape the cells off the plate and collect them in a centrifuge tube. Cells were collected by centrifugation and the pellet was about the size of a mungbean visible to the naked eye. After discarding the fixative solution, a new fixative solution was added and kept at room temperature for 2 h, and then transferred to a 4°C ice bag for transportation. Specimens were kept cold but not frozen during storage or transportation and were analyzed by transmission electron microscopy (HT7700, HITACHI, USA).

MTS and EDU proliferation assays

Adherent cells were plated in 96-well plates until the cell density reached ~70% after 24 h of incubation (4 parallel wells in each group). The medium was removed from the 96-well plate with a pipette and 100 μL of a mixture of complete medium containing 3-(4,5)- dimethylthiahiazo (-z-y1)-3,5-di-phenytetrazoliumromide (MTS; 5 mg/ml, thiazolyl blue; 5:1) were added to each well and gently shaken. Then, the cells were incubated at 37°C, 5% CO2, and 19% O2 for 30 min. The plate was shaken evenly and the color intensity was read at 490 nm wavelength on a microplate reader (Biotek EL×800, USA).

The adherent cells were seeded in 96-well plates and pretreated with Mdivi-1 for 24 h (5 parallel wells in each group). The EDU working fluid (BeyoClick™ EdU Cell Proliferation Kit with Alexa Fluor 647) was prepared with fresh complete RPMI-1640 medium at a concentration of 20 μM. The 2X EdU working fluid was preheated at 37°C and added to a 96-well plate in equal volume and incubated at 37°C, 5% CO2 for 2 h. Next, cells were fixed with 4% paraformaldehyde at room temperature for 15 min and permeated with 0.3% TritonX-100 for another 15 min. Each well was incubated with 60 μL click additive solution for 30 min at room temperature in the dark. Cell nuclei were stained with Hoechst 33342 for 10 min to detect proliferation, which was analyzed using a CellInsight™ CX5 imaging system (Operetta, [PerkinElmer](https://www.perkinelmer.com.cn/), USA).

Analysis of apoptosis by flow cytometry

The PE Annexin V Apoptosis Detection Kit I apoptosis detection kit (BD Bioscience, Franklin Lakes, USA) was used to detect programmed cell death (apoptosis). CNE1-LMP1, HNE2-LMP1, or HONE1-EBV cells were plated in 12-well plates at a 40% density overnight in a 5% CO2, 37°C incubator, and then treated with the Drp1 specific inhibitor, Mdivi-1, at different concentrations or siRNA against Drp1, respectively. After 24 h of treatment with Mdivi-1 or 48 h of Drp1 siRNA, the cells were harvested and washed with PBS. Annexin V binding buffer (100 μL) was added to each well along with 5 μL of 7 ADD and 5 μL of Annexin V-PE and incubated at room temperature in the dark for 30 min. Apoptotic cells were assessed by flow cytometry (FACS Canto II，BD Biosciences) and the data were analyzed using the FlowJo (v.7.6.5; Tree Star Inc., Ashland, OR, USA) software program.

ATP detection

Adherent cells were seeded in 96-well plates until the cell density reached about 80% after 24 h of incubation. The CellTiter-Glo® 2.0 Assay kit (G9242, Promega, WI, USA) was preheated to 22°C before use. The pyrolysis solution and reaction substrate (50 μL) was added to each well and shaken (700 rpm) for 5 min each. Finally, plates were incubated in a 37°C, 5% CO2 incubator for 10 min in the dark. The Medium was removed and the plate was placed on a transparent white board and luminescence was detected using Infinite M200 PRO (Tecan, [Switzerland](https://fanyi.so.com/?src=onebox#Switzerland)).

Measurement of reactive oxygen species (ROS)

To quantify total cellular and mitochondrial ROS, the Mitosox (5 μM) probe was used to stain cells, respectively. Cells were incubated with the probe at room temperature in the dark for 30 min, and then digested using a 0.25% trypsin solution after washing with PBS. The fluorescence intensity was analyzed using a FACS flow cytometer (FACS Canto II, BD Biosciences).

Proximity ligation assay (PLA)

The protein interaction studies were performed with PLA. A Duolink® In Situ Detection Reagents (DUO92101, Sigma, MO, USA) was used according to the manufacturer’s instructions. Cells seeded in four-well chamber slides were washed with PBS, fixed in 4% paraformaldehyde for 30 min, and then permeated in 0.2% Triton X-100 for 20 min. Slides were then blocked with Duolink blocking solution in a pre-heated humidity chamber for 30 min at 37 °C and incubated with the primary antibody to detect Drp1/AMPK (or CDK1) overnight at 4 °C. On the following day slides were incubated with the PLA probes diluted 1:5 in antibody diluents in a pre-heated humidity chamber for 1 h at 37 °C. Subsequent hybridization, ligation, amplification, and detection were performed according to the manufacturer’s protocol. Then the slides were visualized using a confocal microscope (LSM 510 META, Germany).

TUNEL assay

Tumor cells apoptosis levels were measured by TUNEL assay kit (Yeasen, Shanghai, China). Firstly, dewaxing and hydration of tissue sections and incubated with 0.2% Triton X-100 in PBS for 20 min at room temperature. Add 20µg / ml Proteinase K to each section for15 min. Then, prepare the terminal dUTP nick-end labeling reagent by mixing 5 µL of enzyme solution with 45 µL of label solution per slice. Add 50 µL of TUNEL reaction mixture and incubate in an incubator for 1 h at 37˚C prevent from the light. Incubate the cells with Hoechst 33342 (100 ng/mL in PBS) in the dark for 15 min at room temperature. View the slices using a fluorescence microscope or store in the dark.

Extracellular acidification rate (ECAR)

ECAR was measured using the glycolysis stress kit (Luxcel) and analyzed by the XF96 Extracellular Flux analyzer (Seahorse Bioscience). Glycolysis, glycolytic capacity and glycolytic reserve were determined by the sequential addition of 10 mM glucose, 1 PM oligomycin and 50 mM 2-D-glucose.ECAR was normalized to cell protein concentration in each experiment.

CRISPR/Cas9 gene editing

NPC cells were genetically engineered to generate Drp1S616 mutant (S616A) cells using the pRPSGC02 -hDNM1L [sgRNA#1] plasmid and Drp1S637 mutant (S637A) cells using the pRPSGC02 -hDNM1L [sgRNA#2] plasmid. Comprised with lentiCRISPRv2 plasmid (#52961; Addgene), and sgRNA#1 or sgRNA#1for Drp1 (Ser616 or Ser637) were designed using the ATUM gRNA Designer (<https://www.atum.bio/> eComme -rce/cas9/input).


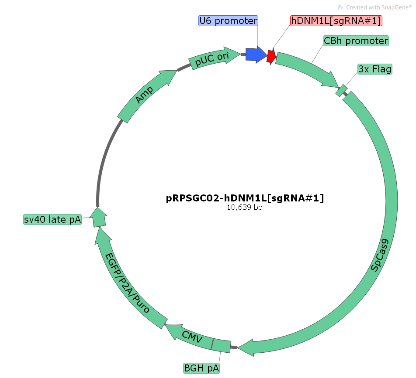

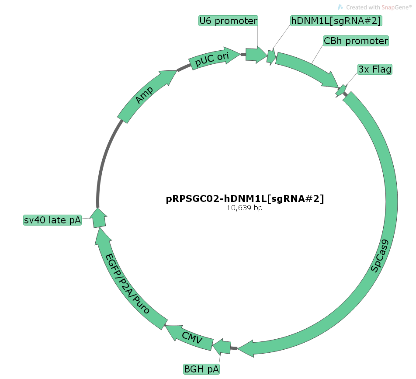


**pRPSGC02-hDNM1L [sgRNA#1] [sgRNA#2]**


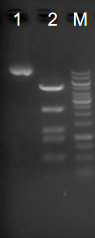

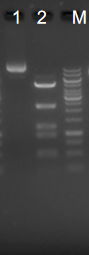

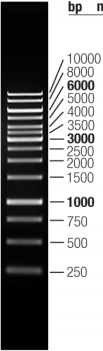


’.


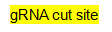
sgRNA#1 sequences ：5’- CCAATTATGCCAGCCAGTCCACA -3’

Wild type genome sequence:

tcaaaagctgaagagttattagcagaagaaaaatcaaaacccattccaattatgccagccagtccacaaaaaggtcatgccgtgaacctgctagatgtggtaagccatgacaatttggtttag

Oligo sequence (S616A)：

tcaaaagctgaagagttattagcagaagaaaaatcaaaacccattccGatAatgccagccGCTccacaaaaaggtcatgccgtgaacctgctagatgtggtaagccatgacaatttggtttag


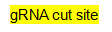
sgRNA#2 sequences ：5’- TTGCACGAAAACTATCTGCTCGG -3’

Wild type genome sequence:

caaaagtaatattttttccccctcatccctatttagccagttcctgttgcacgaaaactatctgctcgggaacagcgagattgtgaggttattgaacgactcattaaatcatattttctcatt

Oligo sequence (S637A)：

caaaagtaatattttttccccctcatccctatttagccagttcctgttgcacgaaaactaGctgcAcgCgaacagcgagattgtgaggttattgaacgactcattaaatcatattttctcatt

**Figure. S1.**

**a**


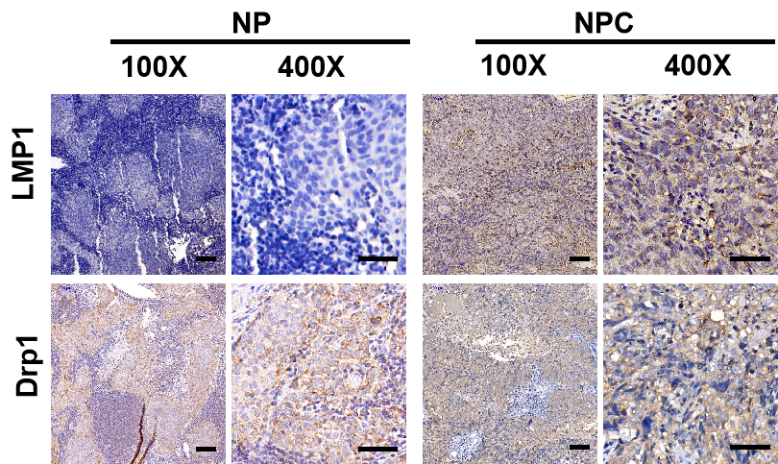


**
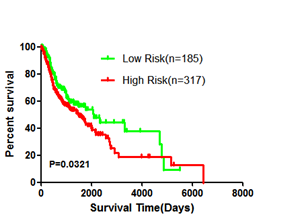
**

**c**

**b**

**Figure S1. EBV-LMP1 relates to the mitochondrial dynamic**

(a) The expression level of DNM1L, Mfn1 and Mfn2 in NPC patients, *** *p* < 0.001. (From the GEO database, https://www. ncbi.nlm.nih.gov/geo/.)
(b) Overall survival rates of head and neck squamous carcinoma patients with low (n = 185) or high (n = 317) mRNA levels of *DNM1L* were estimated with the Kaplan–Meier method by log-rank test (*p* = 0.0321) from the TCGA database.
(c) Representative IHC staining of LMP1 and *Drp1* expression from tissues slices of 26 NPC patients (100x: Scale bar, 100μm; 400x: Scale bar, 50μm).

**Figure. S2.**

**a**

**b**

**Figure S2.** **The level of p-Drp1Ser616 has the function of evaluating clinical stage of patients**

(a) The percentage of specimens displaying TNM stages and p-Drp1Ser616 (P = 0.0002).

(b) The percentage of specimens displaying TNM stages and p-Drp1Ser637 (P=0.087).

**Figure. S3.**


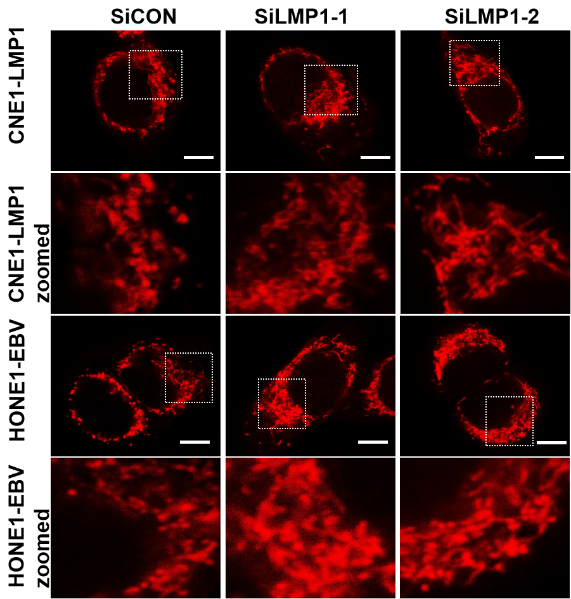

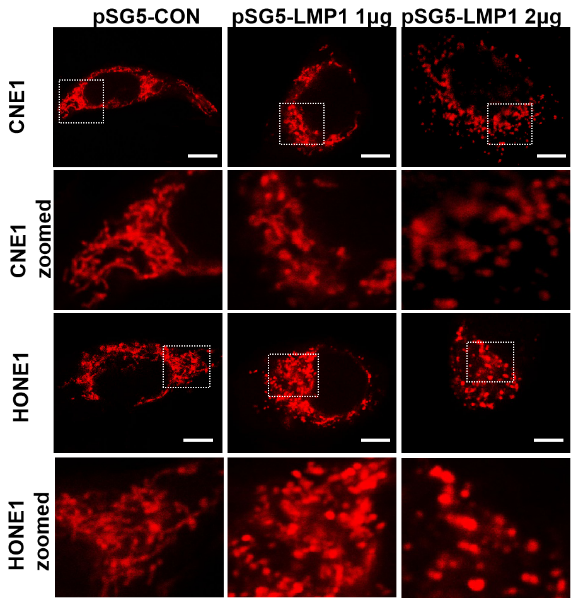


**a**

**b**

**Figure S3. EBV-LMP1 decides mitochondrial morphology**

(a) Mitochondrial morphology of EBV-LMP1 over-expressed cells were observed by staining with Mitotracker Red. Scale bar, 10μm.

(b) Mitochondrial morphology of EBV-LMP1 deleted Cells were determined by confocal microscopy Red: MitoTracker Red (scale bar, 10 μm).

**Figure. S4.**


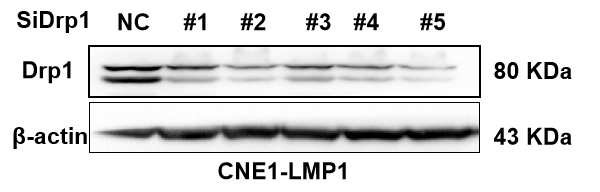


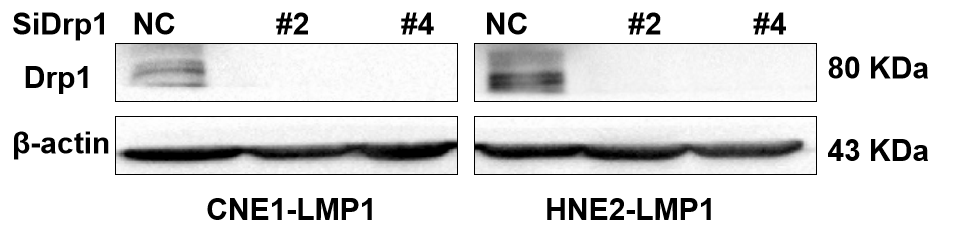


**Figure S4. The effect of Drp1 SiRNA detected by wb**

**Figure. S5.**

**a**


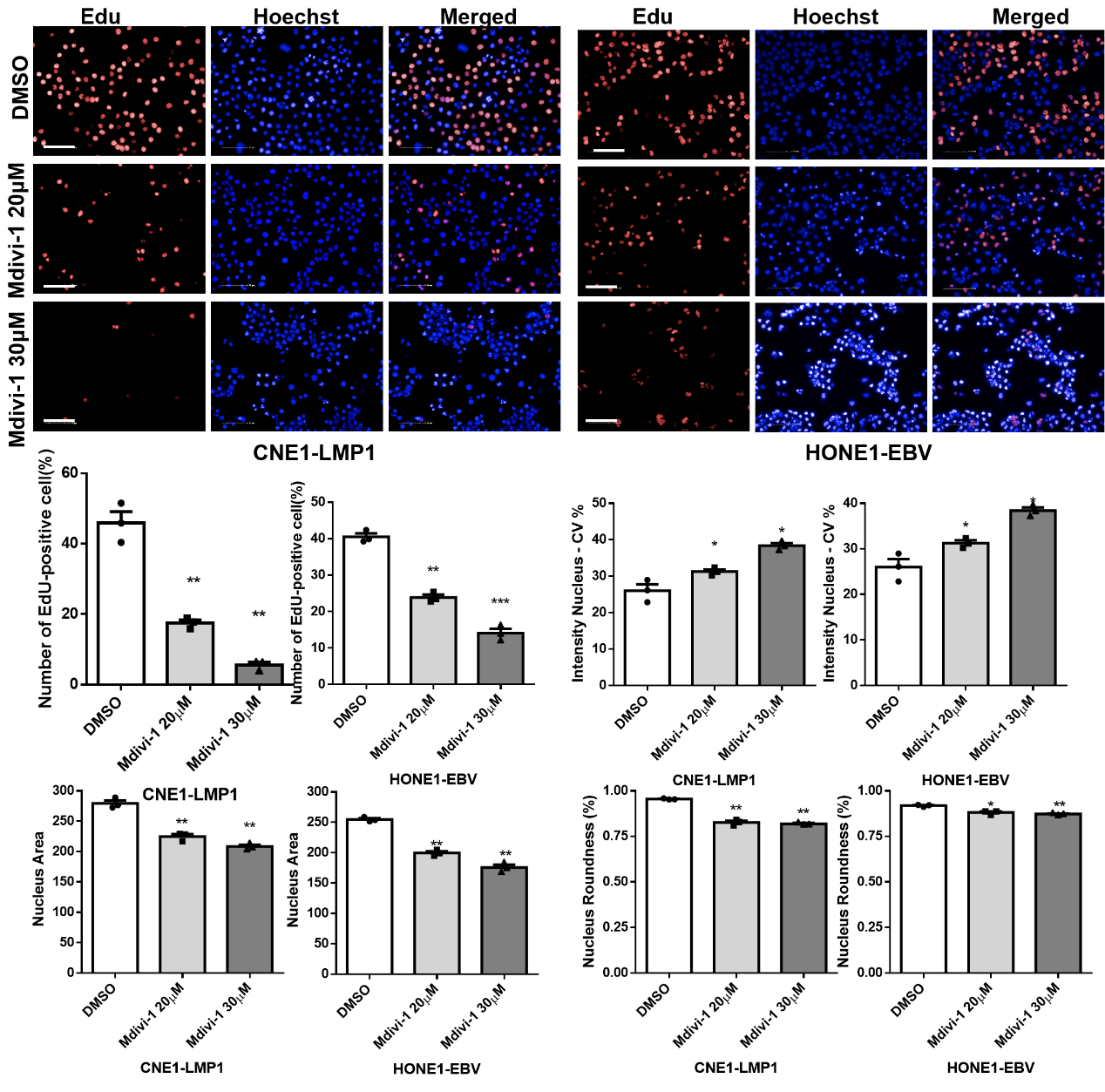


**cD**

**b**

**e**

**d**

**Figure S5. Mdivi-1 inhibits proliferation of NPC cells by decreasing Drp1 activity**

An EdU assay was used to observe the proliferative NPC cells after DMSO and Mdivi-1 treatment for 24 hours.

(a) The EdU-positive proliferative cells (red) were determined by Operetta CLS high-screening imaging system, when nuclei were stained blue (Scale bar, 100μm.)

(b) The number of EdU-positive NPC cells were recorded. Data are shown as mean values ± S.D. (n = 3), (** *p* < 0.01. *** *p* < 0.001.)

(c) The mean nucleus intensity of hoechst 33342 staining (CV %). Data are shown as mean values ± S.D. (n = 3), (**p* < 0.05.)

(d) The mean nucleus area of NPC cells. Data are shown as mean values ± S.D. (n = 3), (** *p* < 0.01.)

(e) The mean nucleus roundness of NPC cells. Data are shown as mean values ± S.D. (n = 3), (**p* < 0.05, ** *p* < 0.01.)

**Figure. S6.**

**a**


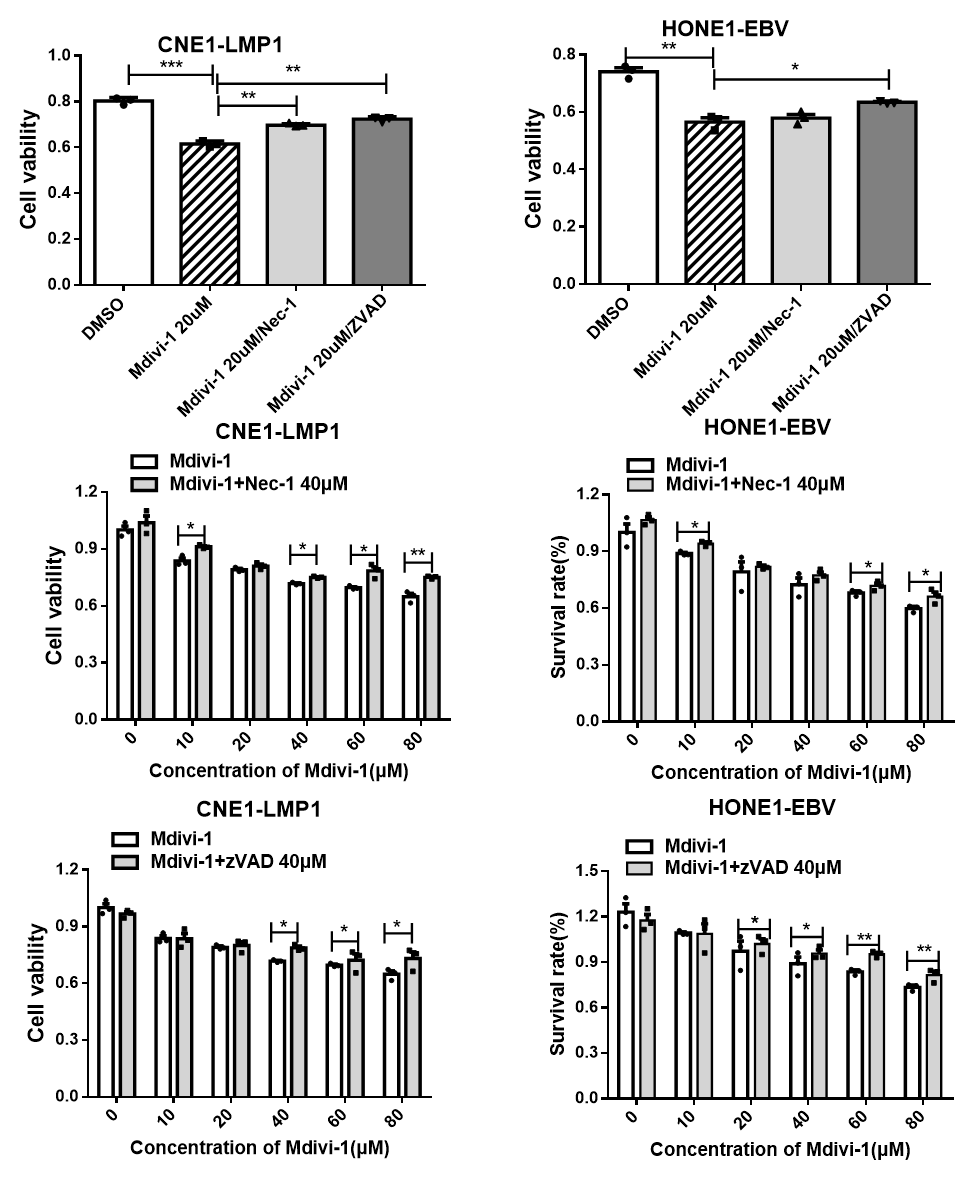


**c**

**b**

**Figure S6. Mdivi-1 leads to cell death**

(a) The necroptosis inhibitor Nec-1 or caspase inhibitor zVAD rescue Mdivi-1-induced cell death. Cells were pretreated with 40 μM Nec-1or zVAD for 30 min, and followed by Mdivi-1 (20 μM) for 24 h. Cell viability was analyzed by the MTS assay. Data are shown as mean values ± S.D. (n = 3), (* *p* < 0.05. ** *p* < 0.01, *** *p* < 0.001.)
(b) CNE1-LMP1 and HONE1-EBV cells were pretreated with 40 μM zVAD for 30 min, and followed by treatment with the indicated concentration of Mdivi-1 for 24 h. Cell viability was analyzed by the MTS assay. Data are shown as mean ± S.D. (n = 3), (* *p* < 0.05. ** *p* < 0.001.)

(c) CNE1-LMP1 and HONE1-EBV cells were pretreated with 40 μM Nec-1 for 30 min, and followed by treatment with the indicated concentration of Mdivi-1 for 24 h. Cell viability was analyzed by the MTS assay. Data are shown as mean values ± S.D. (n = 3), (* *p* < 0.05. ** *p* < 0.001.)

**c**

**a**

**HNE2-LMP1**

**HNE2-LMP1**

**HNE2-LMP1**

**HNE2-LMP1**

**HNE2-LMP1**

**HNE2-LMP1**

**Figure. S7.**

**b**

**d**

**f**

**e**

**h**

**g**

**Figure S7. EBV-LMP1 enhances mitochondrial function by regulating Drp1**

(a-b) Flow cytometry analysis of mitochondrial mass and activity using the MitoTracker Green or Red in NPC cells. Values represent MitoTracker Green/Red intensity as mean values ± S.D. of 3 independent experiments (**p* < 0.05, ***p* < 0.01).
(c-d) Extracellular acidification rate (ECAR) was determined using a Seahorse XF96 analyzer to evaluate the glycolytic flux in LMP1 positive or negative cells.

Values represent mean ± SD of 4 experiments performed in quintuplicates (*p< 0.05, **p< 0.01, ***p< 0.001). Values were normalized to cells protein concentration.

(e-h) HNE2-LMP1 cell was treated with or without mdivi-1 (10μM, 20μM) or transfected with siNC or siDNM1L for 48 h. (e-f) Flow cytometry analysis of mitochondrial mass and activity using MitoTracker Green and Red. Values represent MitoTracker Green and Red intensity mean ± SD of three independent experiments performed. (*p< 0.05, **p< 0.01). (g-h) Effects of Drp1 on adenosine triphosphate (ATP) and ROS. Values represent ATP concentration means ± S.D. of 3 independent experiments (*p< 0.05, **p< 0.01, ***p< 0.001).

**Figure. S8.**

**a**


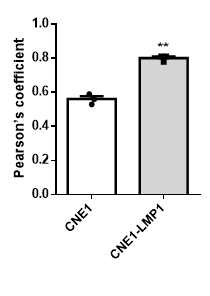


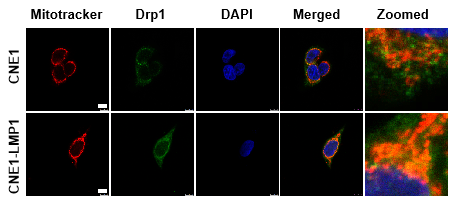


**b**

**c**


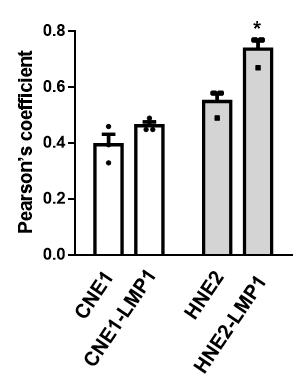


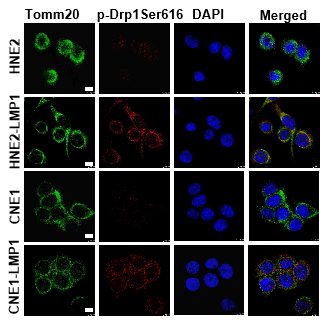


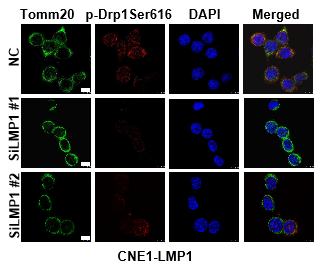


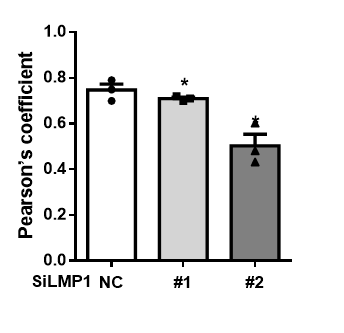


**Figure S8. Additional studies associated with Figure 3.**

(a) IF analysis of Drp1 mitochondrial localization. Red: MitoTracker Red, Green: Drp1. Scale bar, 10μm. Pearson’s coefficient analysis representing relative cellular co-localization of Drp1 overlapping with mitochondria. Images were analyzed using Image J software. (**p < 0.01).

(b-c) Confocal microscopy analysis of subcellular localization of p-Drp1Ser616 in NPC cells (b) or EBV-MP1 deleted Cell (c). Pearson’s coefficient analysis representing relative cellular co-localization of p-Drp1 Ser616 overlapping with mitochondria (*p < 0.05).

**Figure. S9.**


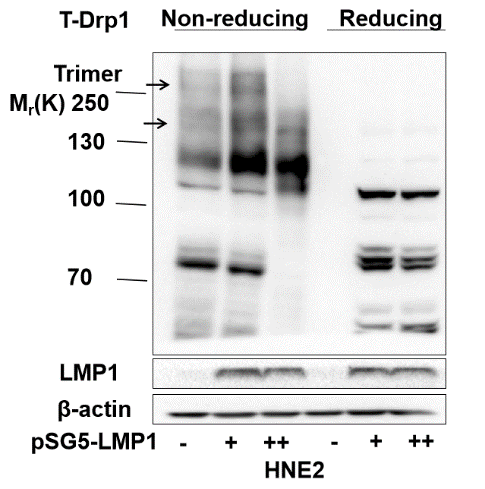

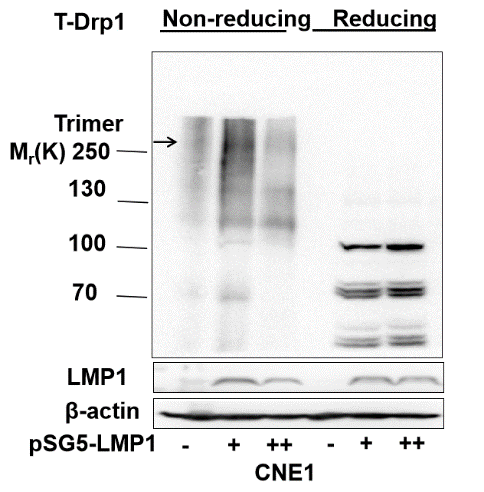


**b**

**a**

**d**

**c**


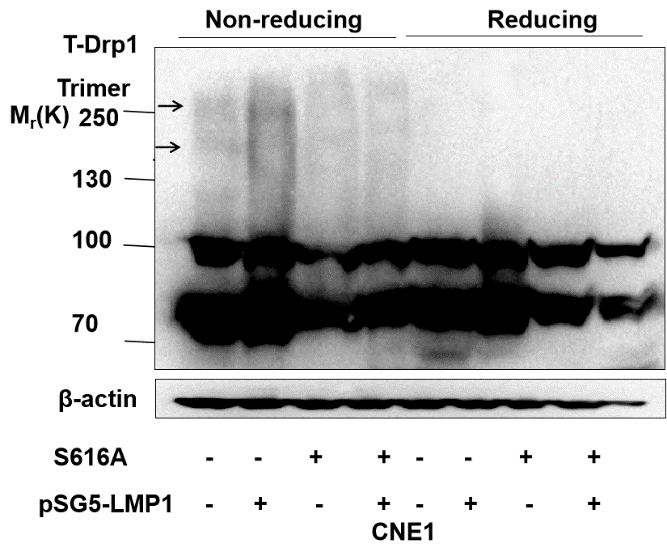


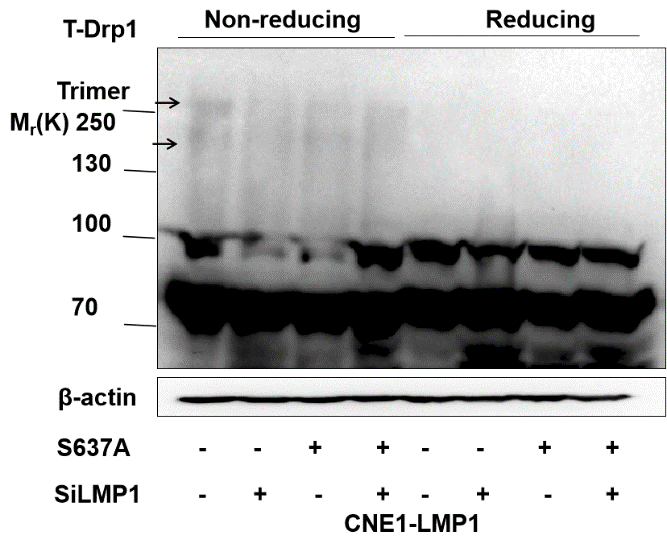


**Figure S9. EBV-LMP1 is the activator for Drp1 oligomerization in NPC cells**

Drp1 oligomers were resolved from monomers by non-reducing SDS-PAGE and specific bands are denoted by arrows.

(a-b) Cell lysates of EBV-LMP1-overexpression cell lines were subjected to Non-reducing SDS-PAGE for detect the Drp1 self-assembly.

(c) Non-reducing SDS-PAGE analysis the change of Drp1 oligomers in CNE1 cell after in endogenous CRISPR/Cas9 mutagenesis Drp1 S616A.

(d) Non-reducing SDS-PAGE analysis the change of Drp1 oligomers in CNE1-LMP1 cell after endogenous CRISPR/Cas9 mutagenesis Drp1 S637A.


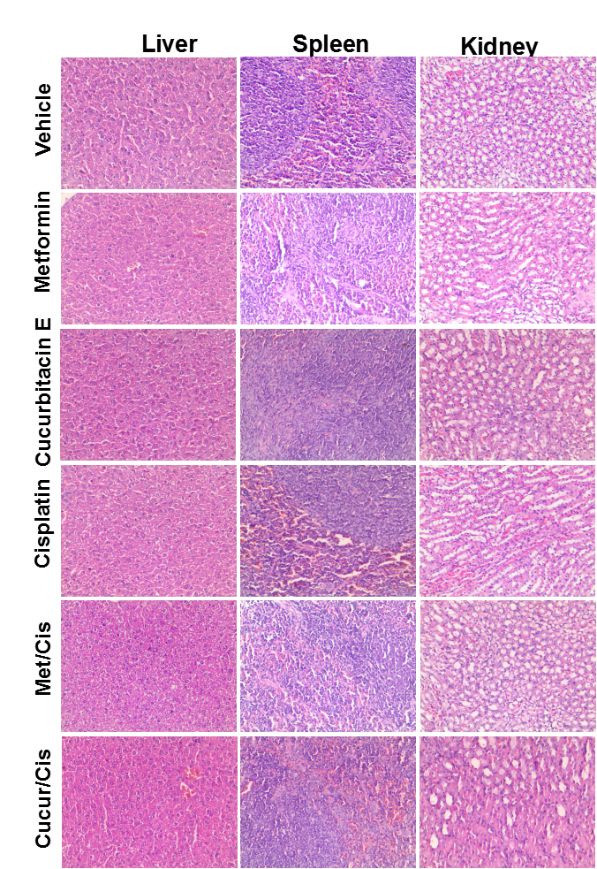
**Figure. S10.**


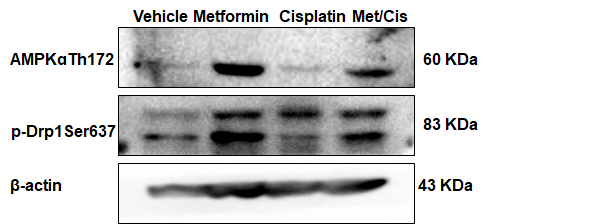


**d**

**a**


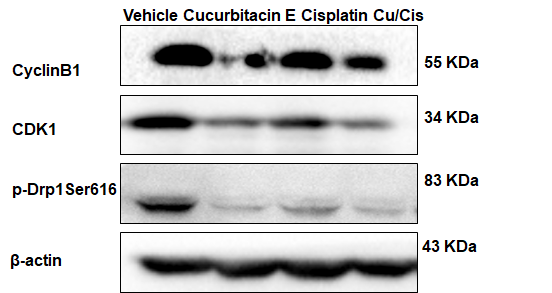


**b**


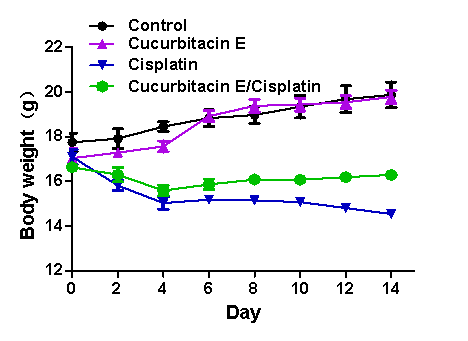

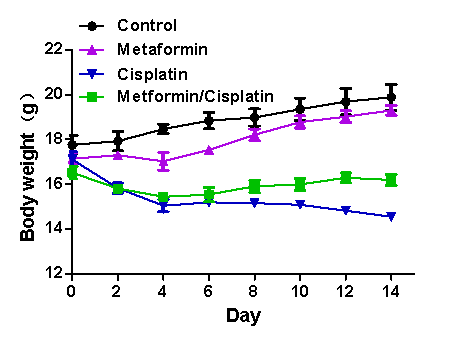


**c**

**Figure S10. Additional *in vivo* animal studies associated with Figure 6.**

(a) Western blot exhibiting the levels of AMPKɑThr172, p-Drp1Ser637, in representative tumor tissue.

(b) The levels of cyclin B1, Cdk1, p-Drp1Ser616 in representative tumor tissue by wb.

(c) The mouse weight curve of the different treatment groups in a CNE1-LMP1 cell xenograft model.

(d) H&E histology of various organs after various treatments for 15 days.

**Table S1.**

**Table S1.** Clinical characteristics and p-Drp1 Ser616 or p-Drp1 Ser637 expression level in 26 NPC patients

| Clinical | p-Drp1 Ser616 | | | *p*-value | p-Drp1 Ser637 | | | *p*-value |
| --- | --- | --- | --- | --- | --- | --- | --- | --- |
|  |  | |  |  |  |  |  |  |
| Characteristics | Low expression | | High expression | | Low expression | High expression | |  |
| Age(yr), ean±SD | 51.57 ± 11.73 | | 58.42 ± 7.33 | 0.08^a^ | 54.39 ± 10.37 | | 55 ± 9.09 | 0.63^a^ |
| Gender |  | |  |  |  | |  |  |
| Male (n) | 12 | | 10 | 0.87^b^ | 19 | | 3 | 0.43^b^ |
| Female (n) | 2 | | 2 |  | 4 | | 0 |  |
| WHO histological classification | | | | | | | | |
| NKUC (UD) | 8 | | 8 | 0.62^b^ | 14 | | 1 | 0.36^b^ |
| NKUC (DF) | 6 | | 4 |  | 9 | | 2 |  |
| ^a^Welch's t test  ^b^Pearson's χ2 test | |  |  |  |  | |  |  |

**Table S2.**

**Table S2.** The relationship between LMP1 and p-Drp1 Ser616 expression in 26 NPC patients

|  |  | p-Drp1 Ser616 | | Total |
| --- | --- | --- | --- | --- |
|  |  | Low | High |  |
| LMP1 | Low | 11 | 2 | 13 |
|  | High | 2 | 11 | 13 |
| Total |  | 13 | 13 | 26 |

*p* = 0.0029, Pearson's χ2 test

**Table S3.**

**Table S3.** The relationship between LMP1 and p-Drp1Ser637 expression in 26 NPC patients

|  |  | p-Drp1 Ser637 | | Total |
| --- | --- | --- | --- | --- |
|  |  | Low | High |  |
| LMP1 | Low | 2 | 11 | 13 |
|  | High | 9 | 4 | 13 |
| Total |  | 11 | 15 | 26 |

*p* = 0.002, Pearson's χ2 test

**Table S4.**

**Table S4.** Clinical characteristics and p-Drp1 Ser616 or p-Drp1 Ser637 expression level in 129 NPC patients

| Clinical | p-Drp1 Ser616 | | *p*-value | p-Drp1 Ser637 | | *p*-value |
| --- | --- | --- | --- | --- | --- | --- |
|  |  |  |  |  |  |  |
| Characteristics | Low expression | High expression | | Low expression | High expression | |
| Age(yr), ean±SD | 48.8 ± 11.41 | 47.4 ± 11.31 | 0.54^a^ | 49 ± 12.01 | 47.2 ± 10.49 | 0.34^a^ |
| Gender |  |  |  |  |  |  |
| Male (n) | 54 | 46 | 0.09^b^ | 53 | 47 | 0.25^b^ |
| Female (n) | 12 | 17 |  | 18 | 11 |  |
| Neck lymph odule Metastasis | | |  |  |  |  |
| Negative | 20 | 15 | 0.3^b^ | 21 | 14 | 0.39^b^ |
| Positive | 46 | 48 |  | 50 | 44 |  |
| TNM |  |  |  |  |  |  |
| Low (**≤**2) | 45 | 26 | 0.0002^b^ | 35 | 36 | 0.09^b^ |
| High (>2) | 21 | 37 |  | 36 | 22 |  |
| ^a^Welch's t test  ^b^Pearson's χ2 test |  |  |  |  |  |  |
